# Cytotoxic Activity of Piperazin-2-One-Based Structures: Cyclic Imines, Lactams, Aminophosphonates, and Their Derivatives

**DOI:** 10.3390/ma14092138

**Published:** 2021-04-22

**Authors:** Jakub Iwanejko, Mahzeiar Samadaei, Matthias Pinter, Daniel Senfter, Sibylle Madlener, Andrzej Kochel, Nataliya Rohr-Udilova, Elżbieta Wojaczyńska

**Affiliations:** 1 Faculty of Chemistry, Wrocław University of Science and Technology, Wybrzeże Wyspiańskiego 27, 50-370 Wrocław, Poland; jakub.iwanejko@pwr.edu.pl; 2 Division of Gastroenterology and Hepatology, Department of Internal Medicine III, Medical University of Vienna, AKH Vienna Währinger Gürtel 18-20, 1090 Vienna, Austria; mahzeiar.samadaei@meduniwien.ac.at (M.S.); matthias.pinter@meduniwien.ac.at (M.P.); nataliya.rohr-udilova@meduniwien.ac.at (N.R.-U.); 3 Department of Pediatrics and Adolescent Medicine, Molecular Neuro-Oncology, Medical University of Vienna, AKH Vienna Währinger Gürtel 18-20, 1090 Vienna, Austria; daniel.senfter@meduniwien.ac.at (D.S.); sibylle.madlener@meduniwien.ac.at (S.M.); 4 Faculty of Chemistry, University of Wrocław, 14 F. Joliot-Curie St., 50-383 Wrocław, Poland; andrzej.kochel@chem.uni.wroc.pl

**Keywords:** aminophosphonates, cyclic imines, cytotoxic activity, Mannich bases, Pudovik reaction, viability assay

## Abstract

*N*-Heterocycles are considered as desirable scaffolds for the development of novel lead compounds for anticancer drug research. Among them, phosphorus-containing amino-derivatives play a crucial role. A series of imines and products of their further reactions with *P*-nucleophiles were obtained starting from vicinal bisamines. Reaction of ethylenediamine and α-carbonyl esters yielded in novel unexpected products, which structures were confirmed by crystallographic measurements. The cytotoxic activity evaluation was done on a variety of cell lines including HUH7, AKH12, DAOY, UW228-2, D283, D425, and U251. Human umbilical vein endothelial cells (HUVECs) were used as control. Two of the tested compounds, bearing TADDOL-derived, and trifluoromethyl substituents showed a significant effect on cell viability, though comparable to nonmalignant cells.

## 1. Introduction

**Figure 1:**
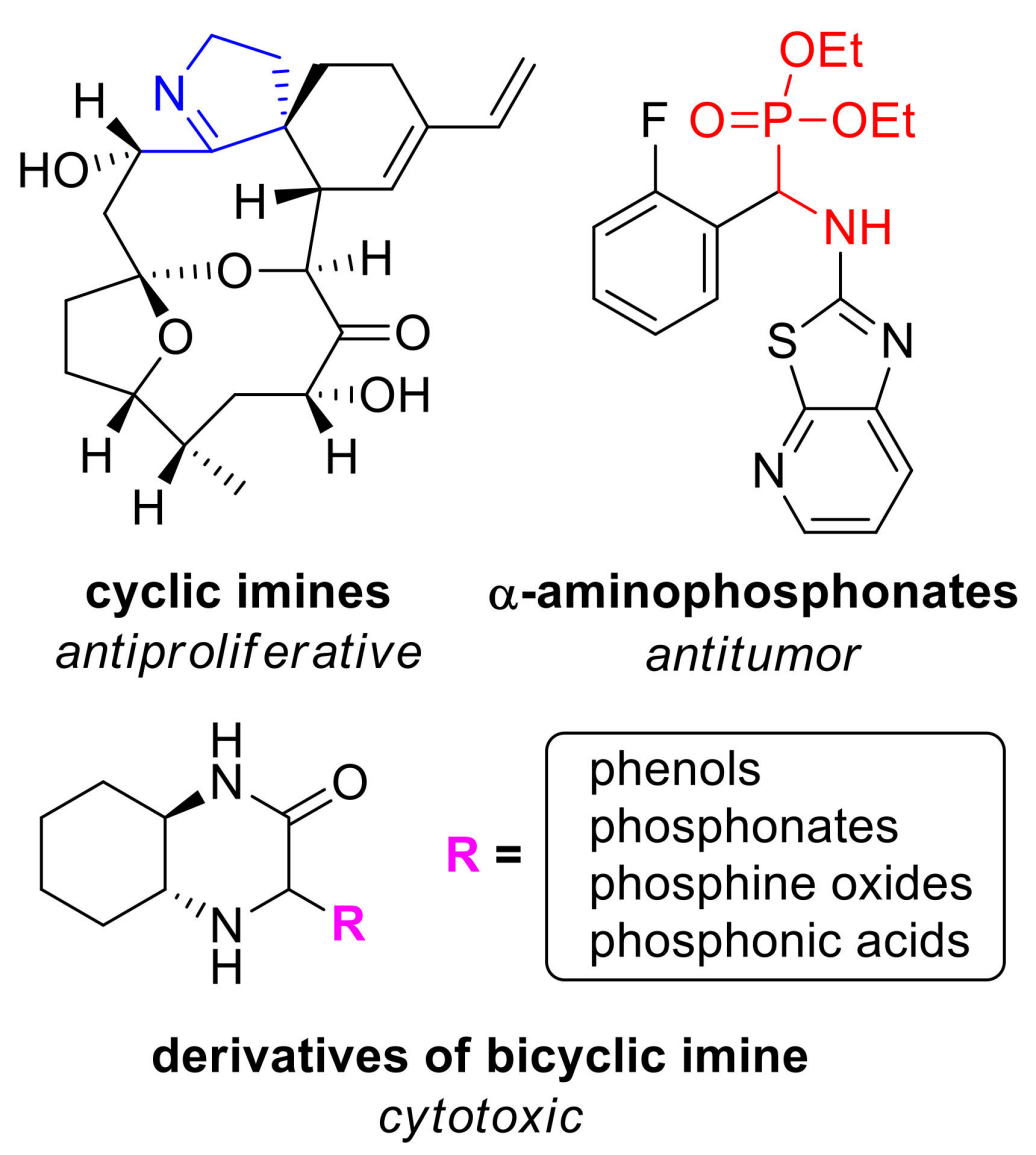
*N*-heterocycles of biological importance. Clockwise from top left: portimine A—marine cyclic imine toxin reducing the growth of various cancer cell lines [18]; α-aminophosphonate posing antitumor activity [19]; general structure of derivatives of bicyclic imine obtained by our group.

Heterocyclic compounds containing nitrogen atom are known for their wide applications in medicinal chemistry [1]. Among the variety of commercially available drugs containing this structural feature, praziquantel—an anthelmintic agent [2], thiamine-vitamin B_1_ and vestipitant—antiemetic and anxiolytic drug [3] are worth mentioning. Naturally occurring and synthetical cyclic imines and their derivatives exhibit antiproliferative and antitumor properties (Figure 1). It is known that coordination complexes bearing aza-heterocyclic ligands found multiple uses in medical diagnostics, therapy, and are popular contrast agents for magnetic resonance imaging (MRI) [4,5]. Reactions of vicinal diamines with dicarbonyl compounds are an efficient and convenient method for the synthesis of *N*-heterocycles.

**Scheme 1:**
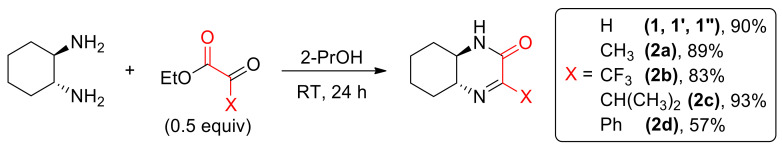
Synthesis of cyclic imines **1**–**2d**.

In our laboratory, we use chiral bicyclic nitrogen–containing compounds as a platform for the preparation of enantiopure compounds which can find an application for the synthesis of drugs and pharmaceuticals. To this end, we obtained a series of enantiopure (N, N), (N, P), and (N, S)-donating ligands and organocatalysts useful in various enantioselective transformations [6,7,8,9], based on the intrinsically chiral skeleton of 2-azanorbornane (2-azabicyclo[2.2.1]heptane) [10]. We established that amino-derivatives inhibited a growth of selected lines of cancer cells [11]. During our research on the application of 1,2-diaminocyclohexane in the synthesis of bicyclic systems, we isolated an enantiopure imine **1** (Scheme 1) and used it in the stereoselective Mannich-type reactions with various nucleophiles, including heterocycles (furan, pyrrole, indole) and substituted phenols [12]. We found that Mannich adducts of di-*tert*-butylphenols to imine **1** showed an interesting antiproliferative activity [13]. Recently, a series of chiral phosphorus derivatives (phosphonates, phosphine oxides, phosphonic acids) of the bicyclic imine have been prepared as well [14,15]. It can be expected that introduction of this functionality into a nitrogen heterocycle can result in a desired biological activity [16]. Indeed, our research proved that incorporation of phosphorus functionality greatly improves antiproliferative activity of *N*-heterocycles obtained by our group [17]. Cytotoxicity of the products were relatively low to normal cells making them promising compounds for drug development. 

Numerous heterocyclic compounds exhibit antiproliferative activity. Typically, they are capable of interacting with a variety of biological targets, e.g., by hydrogen bonding. In particular, they are frequently bound in enzyme pockets.

Our compounds share structural features of a heterocycle, namely octahydroquinoxalinone, and aminophosphonate. Similar structural features are present in other anticancer compounds. For example, quinoxaline derivatives were studied as anticancer agents and apoptotic inducers against three lines of cancer cells, HCT116, HepG1, and MCF-7. The lead compound was found to cause a noticeable disruption in the cell cycle profile, and induced cell cycle arrest at the G2/M phase boundary [20]. On the other hand, in the investigation on antiproliferative activity of aminophosphonates by Huang et al., the authors concluded that the most promising derivative may induce apoptosis of SK-OV-3 cells through mitochondrion pathways [21].

In our previous studies of antiproliferative activity of phosphonic derivatives of octahydroquinoxalin-2(1H)-one [17], the analysis of mechanism of their proliferation inhibition of the MV4-11 cells was performed. We found that most active compounds induce cell apoptosis associated with an increased caspase 3/7 activity. However, various derivatives differed by their specific action, decreasing percentage of cells in different cell cycle phases.

In this contribution, we present the results of an extension of our cytotoxic activity study of various chiral *N*-heterocycles based on vicinal diamines backbone with a focus on imine and aminophosphorus moieties.

## 2. Materials and Methods

**Abbreviations:** dimethyl sulfoxide (DMSO), glioblastoma (GBM), hepatocellular carcinoma (HCC), human umbilical vein endothelial cells (HUVEC), isopropanol (2-PrOH), lactate dehydrogenase (LDH), medulloblastoma (MB), median toxic dose (TD_50_), room temperature (RT), toluenesulfonyl group (tosyl).

### 2.1. Compounds and General Considerations

The reactants and solvents were received from commercial suppliers and used without additional purification. All melting points are uncorrected and determined by the open capillary method using Apotec^®^ Schmelzpunktbestimmer melting point apparatus (WEPA Apothekenbedarf GmbH & Co. KG., Hillscheid, Germany). ^1^H, ^13^C and ^31^P NMR spectra were collected at 25 °C using 400 MHz Jeol 400yh (Jeol Ltd., Tokyo, Japan) and 600 MHz Bruker Avance II 600 instruments (Bruker, Billerica, MA, USA). NMR spectra were measured in CDCl_3_, unless otherwise stated. IR spectra were obtained either as potassium bromide pellets or as liquid films with a Perkin Elmer 2000 FTIR spectrometer (PerkinElmer, Waltham, MA, USA). High-resolution ESI mass spectra were obtained on a Waters LCT Premier XE TOF spectrometer (Waters Corporation, Milford, MA, USA). Optical rotations were determined at 25 °C with an Optical Activity Ltd. Model AA-5 automatic polarimeter (Optical Activity, Ltd., Ramsey, UK); [α]^D^ values are expressed in 10^−1^ deg cm^2^·g^−1^. Silica gel 60 was used for both column chromatography and thin-layer chromatography performed with the Merck plates (F254) which were visualized using UV light and iodine vapors.

#### 2.1.1. Compounds Previously Published

(1*R*,6*R*)-3-oxo-2,5-diazabicyclo[4.4.0]dec-4-ene (1)—[13,17]
(1*S*,6*S*)-3-oxo-2,5-diazabicyclo[4.4.0]dec-4-ene (1′)—[13,17]
±-3-oxo-2,5-diazabicyclo[4.4.0]dec-4-ene (1″)—[13,17]
4-methyl-(1*R*,6*R*)-3-oxo-2,5-diazabicyclo[4.4.0]dec-4-ene (2a)—[15]
4-isopropyl-(1*R*,6*R*)-3-oxo-2,5-diazabicyclo[4.4.0]dec-4-ene (2c)—[15]
4-phenyl-(1*R*,6*R*)-3-oxo-2,5-diazabicyclo[4.4.0]dec-4-ene (2d)—[15]
[4-phenyl-(1*R*,6*R*)-3-oxo-2,5-diazabicyclo[4.4.0]dec-4-yl]-phosphonic acid—[15]
quinoxalin-2(1*H*)-one (3)—[22]
dimethyl-[(1*R*,6*R*)-3-oxo-2,5-diazabicyclo[4.4.0]dec-4-yl] phosphonate (4a)—[14]
diethyl-[(1*R*,6*R*)-3-oxo-2,5-diazabicyclo[4.4.0]dec-4-yl] phosphonate (4b)—[14]
diphenyl-[(1*R*,6*R*)-3-oxo-2,5-diazabicyclo[4.4.0]dec-4-yl] phosphonate (4c)—[14]
dibenzyl-[(1*R*,6*R*)-3-oxo-2,5-diazabicyclo[4.4.0]dec-4-yl] phosphonate (4d)—[14]
(4-((3a*R*,8a*R*)-2,2-dimethyl-6-oxido-4,4,8,8-tetraphenyltetrahydro-[1,3]dioxolo[4,5-e][1,3,2]dioxaphosphepin-6-yl))-(1*R*,6*R*)-3-oxo-2,5-diazabicyclo[4.4.0]decan (4e)—[14]
4-(diphenylphosphoryl)-(1*R*,6*R*)-3-Oxo-2,5-diazabicyclo[4.4.0]decane (4f)—[14]
[(1*R*,6*R*)-3-oxo-2,5-diazabicyclo[4.4.0]dec-4-yl]-phosphonic acid (5a)—[14]
[4-phenyl-(1*R*,6*R*)-3-oxo-2,5-diazabicyclo[4.4.0]dec-4-yl]-phosphonic acid (5b)—[15]
3. -hydroxy-3-(trifluoromethyl)piperazin-2-one (6)—[23]
Herein we present the ^1^H and ^13^C NMR spectra for the first time (see Supporting Information). 3. -phenyl-5,6-dihydropyrazin-2(1*H*)-one (7)—[24]
*N*,*N*’-(ethane-1,2-diyl)bis(2-oxo-2-phenylacetamide) (8)—[25]

In this paper we present the ^1^H and ^13^C NMR spectra for the first time (see Supporting Information). 

#### 2.1.2. New Compounds

4-trifluoromethyl-(1*R*,6*R*)-3-oxo-2,5-diazabicyclo[4.4.0]dec-4-ene (2b). Typical procedure.

(1*R*,2*R*)-1,2-diaminocyclohexane (2.00 mmol, 228 mg, 2.00 equiv) was dissolved in 2-PrOH (4 mL). Ethyl 3,3,3-trifluoropyruvate (1.00 mmol, 0.133 mL, 1.00 equiv) was introduced to the stirred solution and the mixture was stirred for 24 h at room temperature (293 K). The precipitate was filtered off, washed with three portions of 2-PrOH (3 × 2 mL) and air-dried overnight.

Colorless solid (0.183 g, 83%). Mp. 184–185 °C. [α]_D_^20^ −40 (c 0.90, acetone). ^1^H NMR (400 MHz, (CD_3_)_2_DO): δ 7.42 (br. s, 1H), 3.03 (ddd, *J* = 11.2, 9.4, 3.7 Hz, 1H), 2.80–2.86 (m, 1H), 1.84–1.95 (m, 2H), 1.68–1.74 (m, 2H), 1.18–1.42 (m, 4H). ^13^C NMR (100 MHz, (CD_3_)_2_DO): δ 164.9, 123.4 (q, *J* = 285.6), 82.7 (q, *J* = 28.9), 57.3, 53.5, 30.0, 29.8, 24.0, 23.6. IR (KBr): 3351, 3211, 3108, 2949, 2888, 2869, 1679, 1652, 1477, 1427, 1192, 1155, 1061, 1000, 806 cm^−1^. HRMS (ESI+, m/z): calcd. for C_9_H_12_F_3_N_2_O ([M + H]^+^), 221.0902, found, 221.0910.

3,8-Diphenyl-1,4,7,10-tetraazacyclododeca-3,7-diene-2,9-dione (9); Typical procedure

Ethylenediamine (10.0 mmol, 601 mg, 0.668 mL, 1.00 equiv) was dissolved in 2-PrOH (20 mL). Ethyl 2-oxo-2-phenylacetate (10.0 mmol, 1.78 g, 1.00 equiv) was then added and the resulting mixture was stirred at 293 K for 48 h. After removal of solvent in vacuo, the products were isolated by chromatography on silica gel column (eluent: CH_2_Cl_2_/MeOH 80:20 *v*/*v*).

Colorless solid (0.045 g, 13%). Mp. 291–293 °C. ^1^H NMR (600 MHz, DMSO-d_6_): δ 8.70 (br. s, 2H), 7.72–7.74 (m, 4H), 7.46–7.52 (m, 6H), 4.08 (br. s, 4H), 3.40 (br. s, 2H), 3.03 (br. s, 2H). ^13^C NMR (151 MHz, DMSO-d_6_): δ 167.3 (2C overlapped), 165.9 (2C overlapped), 135.1 (2C overlapped), 131.3 (2C overlapped), 129.0 (4C overlapped), 127.7 (4C overlapped), 55.6 (2C overlapped), 37.2 (2C overlapped). HRMS (ESI+, *m*/*z*): calcd. for C_20_H_21_N_4_O_2_ ([M + H]^+^), 349.1664, found, 349.1659. 

Hexahydroimidazo[1′,2′:3,4]imidazo[1,2-*a*]pyrazine-5,10(4a*H*,6*H*)-dione (10). Typical procedure.

Ethylenediamine (0.668 mL, 10.0 mmol) was dissolved in ethanol (20 mL). To the stirred solution ethyl glyoxalate (~50% in toluene, 2.10 mL, 10.0 mmol, 1.00 equiv) was introduced, followed by stirring for 48 h at room temperature (293 K). Solvents were evaporated under vacuo, and the products were purified on a silica gel column (eluent: CH_2_Cl_2_/MeOH 80:20 *v*/*v*).

Colorless solid (0.153 g, 78%). Mp. 185–187 °C. ^1^H NMR (500 MHz, D_2_O): δ 4.68 (d, *J* = 1.5 Hz, 1H), 4.44 (d, *J* = 1.0 Hz, 1H), 3.73–3.81 (m, 1H), 3.24–3.37 (m, 3H), 3.09 (ddd, *J* = 12.5, 5.5, 1.0 Hz, 1H), 3.01 (ddd, *J* = 12.2, 6.0, 1.5 Hz, 1H), 2.87 (dq, *J* = 12.0, 5.5 Hz, 1H) 2.43 (dq, *J* = 11.5, 6.0 Hz, 1H). ^13^C NMR (100 MHz, DMSO-d_6_): δ 169.5, 168.5, 79.4, 75.9, 55.4, 43.8, 39.9, 37.2. IR (KBr): 3433, 3319, 3224, 2933, 2882, 1690, 1667, 1473, 1328, 1137, 1084, 975, 806 cm^−1^. HRMS (ESI+, *m*/*z*): Found, 197.1032; calcd. for C_8_H_13_N_4_O_2_ ([M + H]^+^), 197.1039.

### 2.2. Single Crystal X-ray Structure Determination of 8, 9 and 10

Crystallographic measurements for 8, 9 and 10 were collected at 100(2) K with Κ-geometry diffractometers: Xcalibur Ruby and KM4 Sapphire 2 CCD detector (for 8) equipped with an Oxford Cryosystems cooler, using Mo-Kα radiation (λ = 0.71073 Å, graphite monochromator). CrysAlisPro software was used for data collection, cell refinement, data reduction and analysis [26]. CrysAlisPro was also applied for introduction of analytical absorption correction. SHELXS [27] was used for solving crystal structures, and refinement on F^2^ by a full-matrix least squares technique was performed with SHELXL-2014/16 [28] using anisotropic thermal parameters for all the ordered non-H atoms. H atoms were repositioned in their calculated positions in the final refinement cycles, and treated as riding atoms, with C-H = 0.95–0.98 Å, and with U_iso_(H) = 1.2U_eq_ (C) for CH,CH_2_. All figures were prepared using DIAMOND program [29].

CCDC (2057703-10, 2057704-9, 2062566-8) contain the supplementary crystallographic data for this paper. These data can be obtained free of charge via www.ccdc.cam.ac.uk/data_request/cif, by e-mailing data_request@ccdc.cam.ac.uk, or by contacting the Cambridge Crystallographic Data Centre, 12 Union Road, Cambridge CB2 1EZ, UK; fax: +44(0)1223-336033.

### 2.3. Biological Activity Analysis

#### 2.3.1. Cell Lines and Culture Conditions

The human HCC cell line, HUH7 was purchased from ATCC (LGC standards GmbH, Wesel, Germany). The Austrian human HCC cell line AKH12 has been well characterized [30] and was kindly provided by Prof. Bettina Grasl-Kraupp. HUH7 cells were cultured in DMEM-Dulbecco’s Modified Eagle Medium (Thermo Fisher Scientific, Waltham, MA, USA) containing 10% heat inactivated fetal bovine serum (FBS) (Sigma-Aldrich, St. Louis, MO, USA), 1% penicillin-streptomycin (10,000 U/mL) (Thermo Fisher Scientific, Waltham, MA, USA), and 1% Minimum Essential Medium Non-Essential Amino Acids (100×) (Thermo Fisher Scientific, Waltham, MA, USA). AKH12 cells were cultured in RPMI-1640 Medium (Sigma-Aldrich, St. Louis, MO, USA) containing 10% heat inactivated FBS. DAOY and D283Med (short D283) medulloblastoma cells were purchased from ATCC (LGC standards GmbH, Wesel, Germany). D425Med (short D425) and UW228-2 were provided by Thomas Ströbel from the Institute of Neurology Medical University of Vienna. The cells were cultured in Dulbecco’s Modified Eagle Medium (Sigma-Aldrich, St. Louis, MO, USA) containing 10% FBS (Sigma-Aldrich, St. Louis, MO, USA), 2% L-Glutamine (Sigma-Aldrich, St. Louis, MO, USA), and 0.2% Normocin (Sigma-Aldrich, St. Louis, MO, USA). U-251 MG (short U251) glioblastoma cell line was purchased from Sigma-Aldrich and cultured in Dulbecco’s Modified Eagle Medium (Sigma-Aldrich, St. Louis, MO, USA) containing 10% FBS (Sigma-Aldrich, St. Louis, MO, USA), 2% L-Glutamine (Sigma-Aldrich, St. Louis, MO, USA), and 0.2% Normocin (Sigma-Aldrich, St. Louis, MO, USA). HUVEC cells were provided by Brigitte Winter from the Department of Surgery Medical University of Vienna and were grown in Endothelial Cell Growth Medium-2 BulletKitTM (Lonza, Basel, Switzerland) under standard tissue culture condition. All cells were incubated at 37 °C in a 5% CO_2_ atmosphere.

#### 2.3.2. Neutral Red Cell Viability Assay (Adherent Cell Lines)

Neutral red assay was applied for cell viability determination as described previously [31]. 3 × 10^4^ cells were placed in 24-well plates and grown for 24 h prior to treatment with compounds. After treatment for 24 h, cells were incubated at 37 °C with neutral red dye at 50 µg/mL concentration in serum free medium for 2 h. Cells were washed twice with PBS and incubated with 1% CH_3_COOH in 70% C_2_H_5_OH. Concentration of the dye was determined photometrically at 562 nm. The percentage of survival was expressed as ratio of survival against cells treated with vehicle (DMSO; indicated as 0 µM concentration). TD_50_ was defined as the concentration of compound required to eliminate half of cells after a specified test duration. 

#### 2.3.3. CellTiter-Blue Cell Viability-Assay (Suspension Cell Line)

One day before treatment 2 × 10^4^ D425Med cells were seeded in 24-well plates and incubated at 37 °C in a humidified atmosphere containing 5% CO_2_. The cells were treated with investigated piperazin-2-one derivatives for 24 h. A cell viability assay was performed using the CellTiter Blue Reagent (Promega, Fitchburg, WI, USA) in agreement with the manufacturer’s protocol. As the method for the cell viability was changed for this cell line, we applied the same method for HUVEC cell line for comparison. Three independent experiments were performed. The percentage of survival was expressed as ratio of survival against of DMSO-treated cells (indicated as 0 µM concentration).

#### 2.3.4. Determination of Lactate Dehydrogenase (LDH)

The release of lactate dehydrogenase into culture media was determined by a cytotoxicity detection kit from Roche Diagnostics according to the manufacturer’s instruction. Triton X 100-lysed cells with the maximum LDH activity were considered as a 100% positive control. DMSO used as the vehicle control.

## 3. Results and Discussion

### 3.1. Chemistry

Chiral, enantiomerically pure imine (1*R*,6*R*)-**1** was prepared from (1*R*,2*R*)-1,2-diaminocyclohexane (DACH) and ethyl glyoxylate as described previously [13] (Scheme 1). Using appropriate reactants, compound **1** was also prepared as (*S*,*S*) enantiomer (**1′**) as well as a racemate (**1″**). A series of enantiopure 4-substituted derivatives **2a**–**2d** were obtained from DACH and ethyl esters of ketoacids [15].

**Scheme 2:**
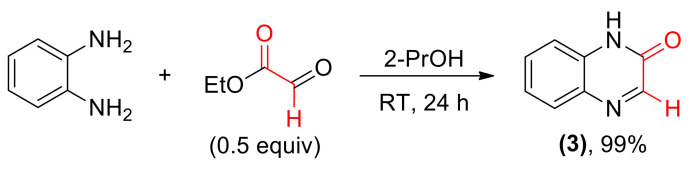
Synthesis of aromatic cyclic imine **3**.

Also an achiral quinoxalin-2(1*H*)-one **3** was prepared from o-phenylenediamine and ethyl glyoxylate (Scheme 2). 

**Scheme 3:**
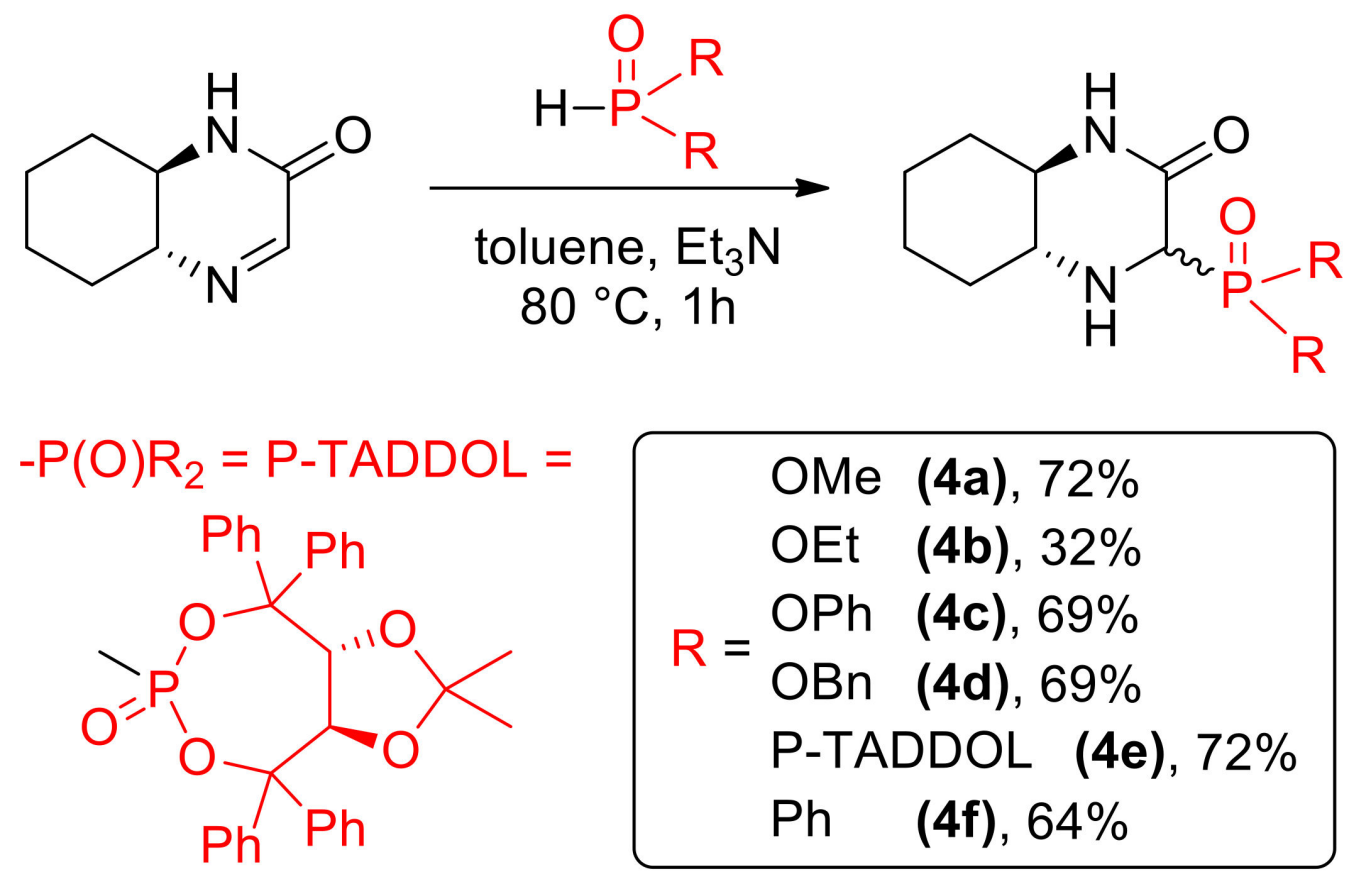
Nucleophilic C=N addition of phosphorus nucleophiles.

As previously described, cyclic imine **1** reacts with a variety of nucleophiles (Scheme 3). Addition of phosphorus derivatives proceeded with generally high yields, but low diastereoselectivity, yielding aminophophonates **4a**–**4e** and phosphine oxide **4f** as diastereomeric mixtures [14]. Isomers of **4e** were separated using column chromatography, however further studies showed that epimerization of those stereoisomers slowly occurs in solution. For this reason, we used mixtures of epimers in cytotoxicity testing. 

**Scheme 4:**
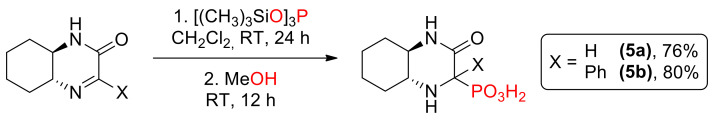
Synthesis of aminophosphonic acids **5a** and **5b**.

In addition, two phosphonic acids **5a** [14] and **5b** [15] were prepared via formation of silyl esters followed by methanolysis (Scheme 4); this time, the reaction yielded mainly one (in case of **5a**, dr 70:30) or exclusively one diastereomer (for **5b**).

**Scheme 5:**
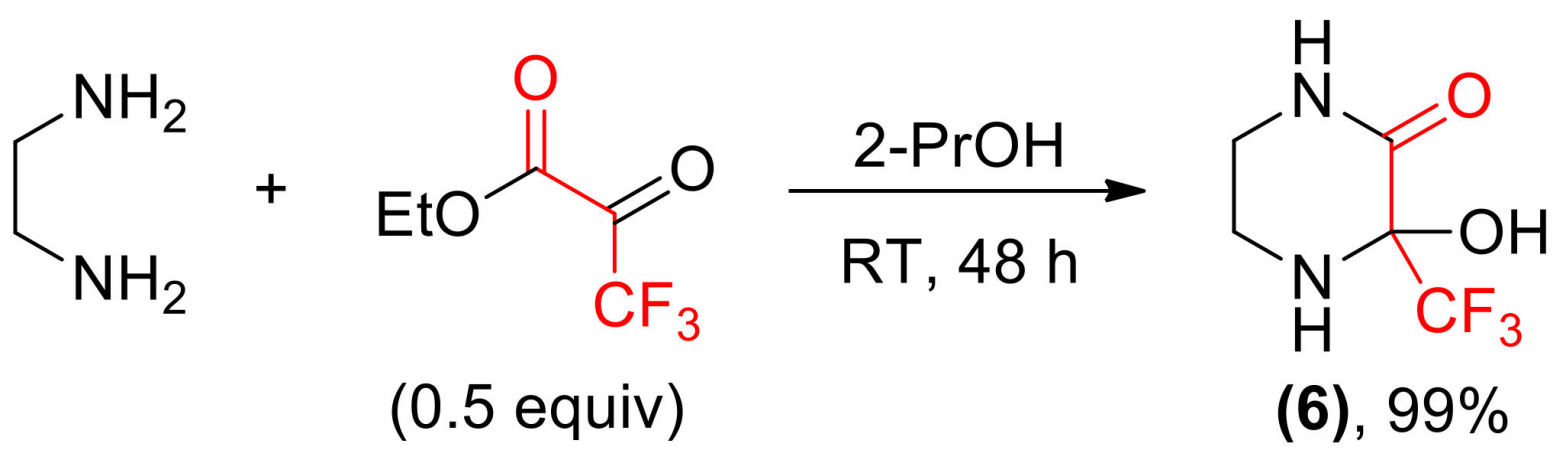
Synthesis of imine hydrate **6**.

We turned our attention towards the synthesis of monocyclic compounds, such as 3-hydroxy-3-(trifluoromethyl)piperazin-2-one **6**, by conducting the reactions of ketoesters with ethylenediamine. The results of these preparations were found dependent on the structure of carbonyl substrate. The presence of highly electrophilic –CF_3_ moiety led to the formation of hydrate form of imine in a quantitative yield (Scheme 5). 

**Scheme 6:**
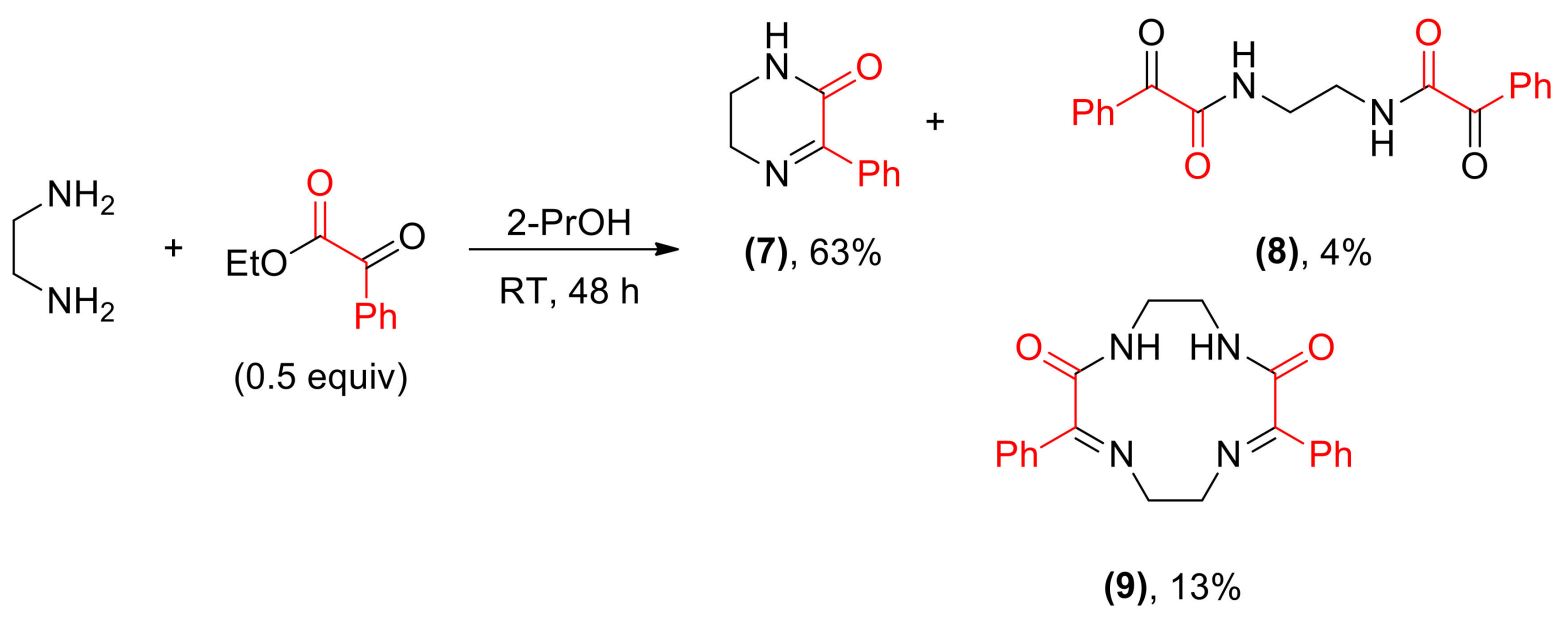
Reaction between ethylenediamine and benzoylformate.

**Figure 2:**
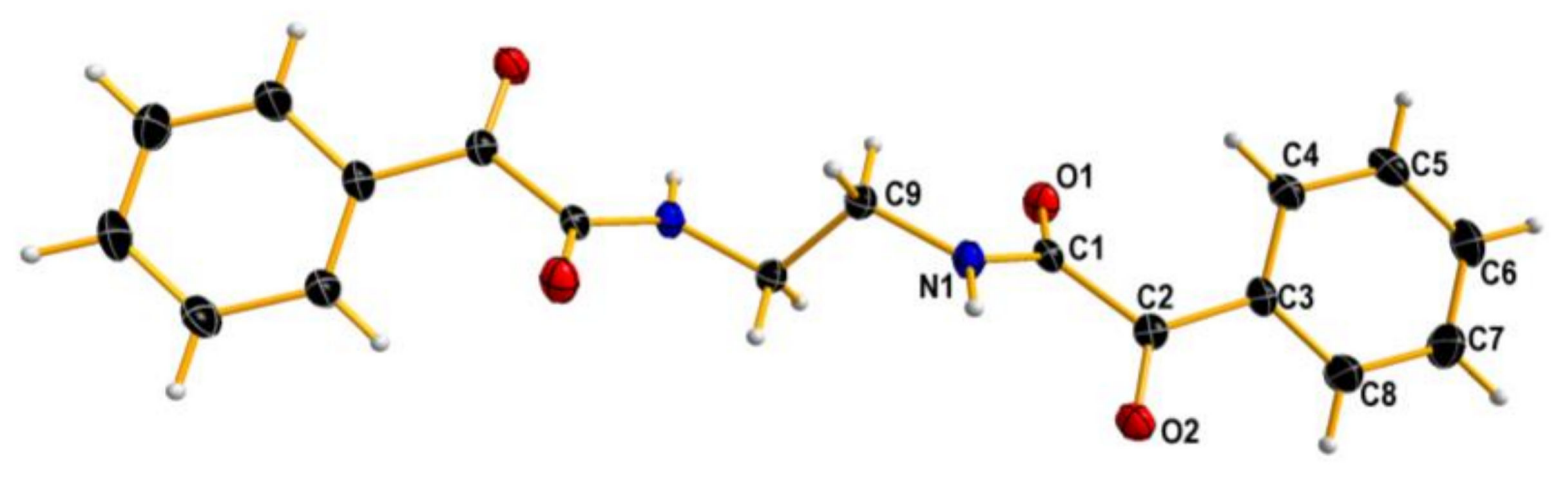
X-ray structure of **8**. Displacement ellipsoids are shown at the 50% probability level. Hydrogen atoms are depicted as small spheres of arbitrary radii.

**Figure 3:**
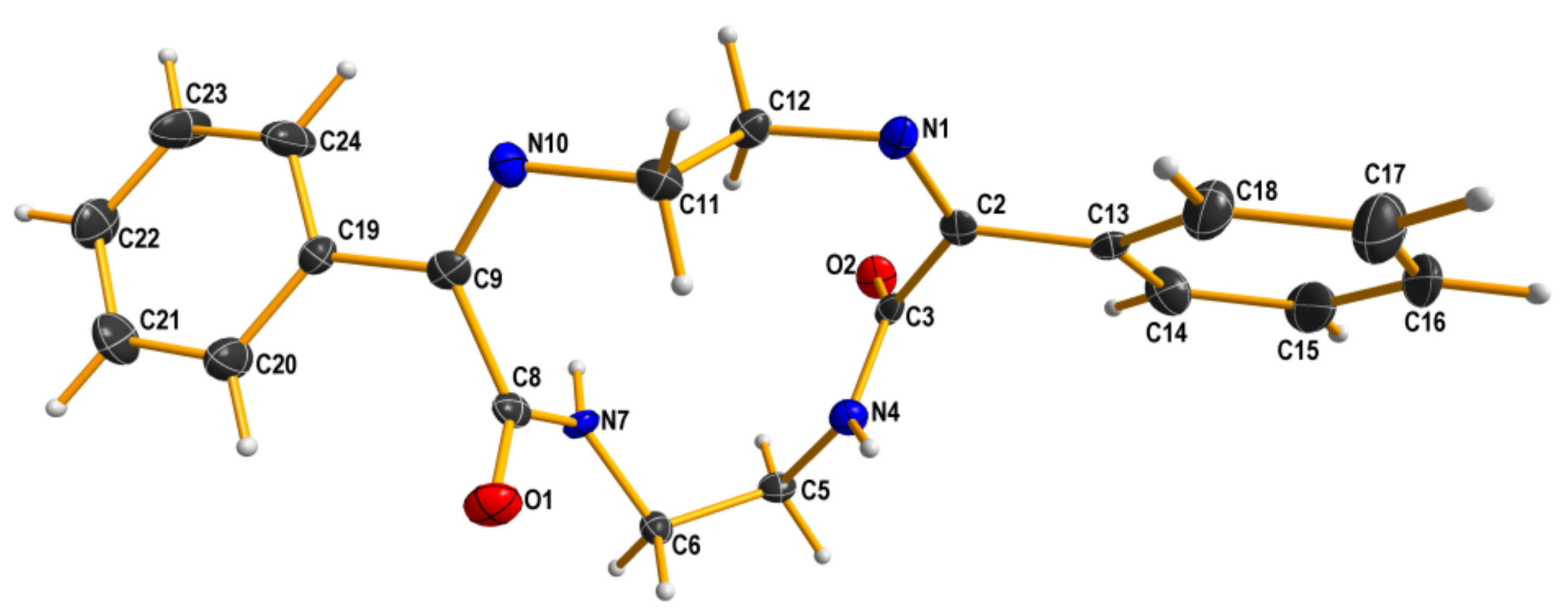
X-ray structure of **9**. Displacement ellipsoids are shown at the 50% probability level. Hydrogen atoms are drawn as small spheres of arbitrary radii.

Reaction of ethylenediamine and ethyl benzoylformate yielded a complex mixture from which three main products: a cyclic imine **7**, a C_2_-symmetrical amide **8** and a macrocyclic compound **9** were isolated (Scheme 6). The structure of two latter products in the solid state was proven by X-ray measurements (Figure 2 and Figure 3). 

**Figure 4:**
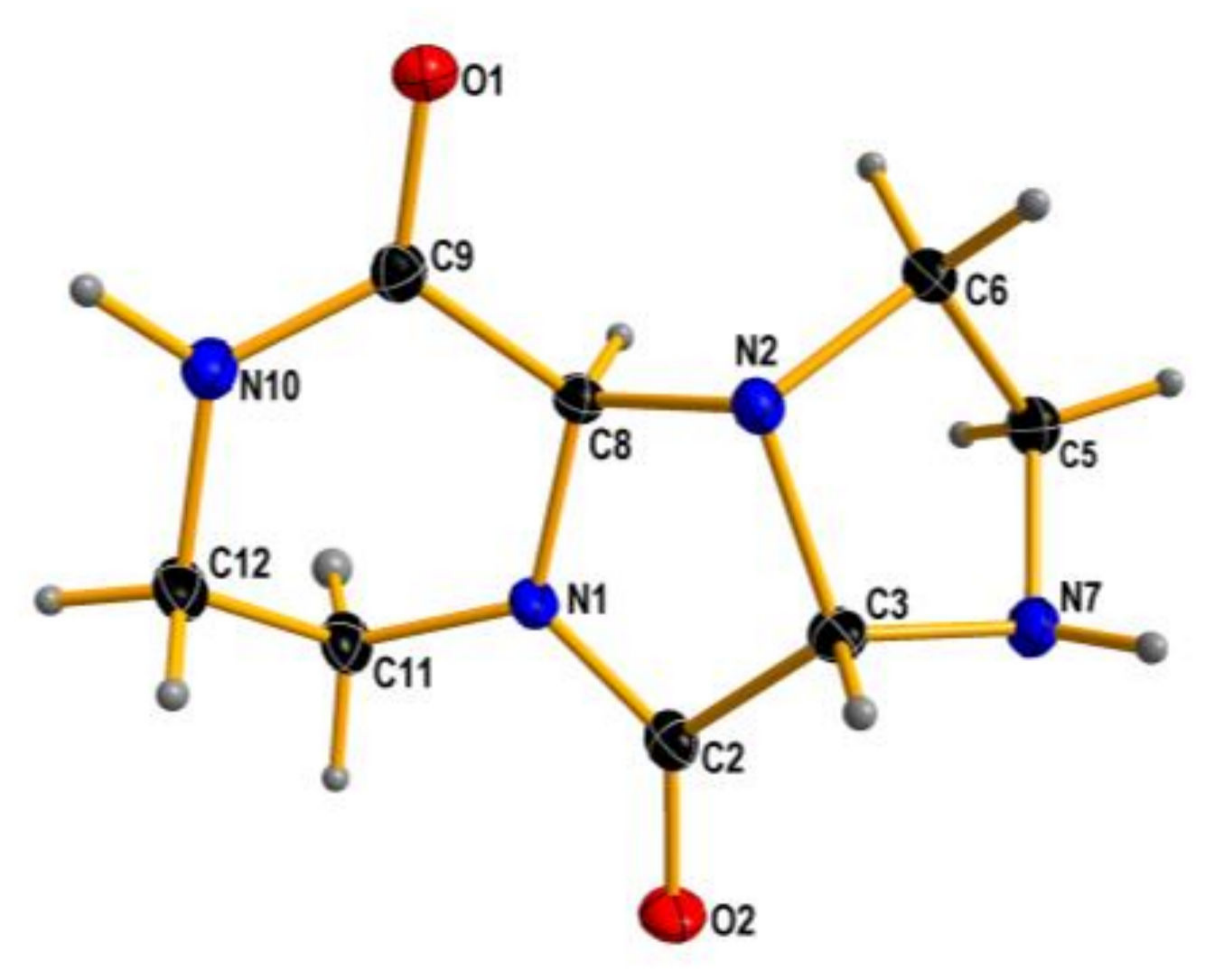
X-ray structure of **10**. Displacement ellipsoids are displayed at the 50% probability level. Hydrogen atoms presented as small spheres of arbitrary radii.

To our surprise, ethylenediamine reacted with ethyl glyoxylate in a completely different manner: instead of analogs of compounds **6**–**9**, a tricyclic dilactam **10** was formed. This unexpected product can pose as an useful scaffold for organic synthesis. The structure of compound **10** in chloroform solution was established by 2D NMR techniques, and was identical with solid state structure obtained from X-ray measurements (Figure 4). It is also worth noting that the formation of the tricyclic product is diastereoselective—both stereocenters have the same configuration. Certainly, one cannot expect enantioselective formation of a specific stereoisomer, and the compound is racemic.

A similar aza-heterocyclic scaffold was obtained by Li and co-workers via a five-component cyclization of *N*-tosyl-ethylenediamine with glyoxal in methanol [32]. The authors noticed the relationship between the stoichiometry of starting materials and the structure of the obtained *N*-heterocycle in the presence of transition metal salts as catalysts. Various molar ratio directed the reaction towards synthesis of monocyclic imine, tricyclic tetraamine or double bicyclic structure. Our approach is quite simpler, and the use of catalysts was not necessary. We observed a dependence between ethyl glyoxylate form used and the yield of tricyclic product. As seen in Table 1, the lowest yield was obtained using glyoxyate prepared in situ by cleavage of diethyl tartrate with periodic acid in diethyl ether (entry 1) [12]. A slight improvement was observed when this starting material was obtained immediately before the synthesis, isolated and used right after (entry 2). The use of a commercially available polymeric form (47% solution in toluene) led to higher yields (entries 3–5). By elongation of the reaction time, we were able to obtain compound **10** with a relatively good efficiency. 

Using a presented set of reactions, we obtained a series of various nitrogen-based heterocycles for cytotoxic activity tests. Structural variations of the compounds was applied to broadly evaluate and discuss the structure-activity relationship. Due to the poor solubility in DMSO, compounds **9** and **10** were excluded from the cellular in vitro assays.

### 3.2. Cytotoxic Effect of Tested Compounds

#### 3.2.1. Primary Evaluation of the Anti-Cancer Activity of the Compounds

The general approach to the evaluation of biological activity of investigated compounds followed the one described in our previous study on sulfonamides based on 2-azanorbornane skeleton [33]. All compounds were dissolved in dimethyl sulfoxide (DMSO). In our experimental design, all compounds that reduced cells viability by more than 50% at 50 µM concentration after 24 h were considered as positive hints and were eligible for lactate dehydrogenase (LDH) cytotoxicity assay. To assess the anti-cancer activity of compounds, we have tested hepatocellular carcinoma (HCC), medulloblastoma (MB), and glioblastoma (GBM) cell lines. Further, to compare the effects of selected compounds on non-malignant primary cells, we used human umbilical vein endothelial cells (HUVEC).

#### 3.2.2. Impact of Compounds on HCC Cell Lines

HCC accounts for a vast majority of liver cancer and is among the leading causes of cancer-related mortality with the highest incidence in Northern and Western Africa and Eastern and South-Eastern Asia [34]. The introduced 50 µM of two compounds, **4e** and **6** reduced cell viability below TD_50_ in HUH7 and AKH12 cell lines (Figures S7A and S9A). The cytotoxicity of mentioned compounds was measured by the LDH release in a dose-dependent manner (Figures S7B and S9B). The efficacy of tested compounds was compared between HCC cells and HUVEC cells. Our results showed a significant activity of both compounds. However, no reduction as compared to HUVEC cells was observed (Figures S8 and S10 and Table 2).

#### 3.2.3. Compounds and MB Cell Lines

MB considered being the most common malignant brain tumor, comprises a biologically heterogeneous group of embryonal tumor with an estimated 5000–8000 cases per year worldwide [35]. In current classification MB divided to four molecular subgroups namely, wingless (WNT), sonic hedgehog (SHH), Group 3, and Group 4 each of which is associated with different genetic alteration, age at onset and prognosis [36]. Among the MB cells that we tested, DAOY and UW228-2 belong to SHH subgroup whereas D425 correspond to Group 3, and D283 exhibits features of both Group 3 and Group 4 [37]. We observed that compounds **4e** and **6** with 50 µM concentration reduced cell viability in all 4 cell lines (Figures S11A, S13A, S15A and S17A). LDH release indicating the loss of membrane integrity was shown in Figures S11B, S13B, S15B, and S17B. The TD_50_ values for the investigated compounds in all MB cell lines and HUVEC cells are shown in Table 2 and Table 3. No significant effects were observed as compared to HUVEC (Figures S12, S14, S16, and S18).

#### 3.2.4. Compounds and Glioblastoma Cell Line

Despite aggressive therapy glioblastoma is still associated with a dismal prognosis in almost all adult patients [38]. U251 glioblastoma cell line was subjected to 50 µM of studied derivatives. Compounds **4e** and **6** decreased cell viability by more than 50% (Figure S19A). The LDH cytotoxicity assay indicating necrotic cell death was performed in a dose-dependent manner (Figure S19B). However, there was no reduction in U251 cells viability as compared to HUVEC (Figure S20). Table 2 presents the TD_50_ values of tested compounds on U251 and HUVEC cell lines.

The results of our study show that most of the investigated compounds have a negligible effect on the cell viability of the lines tested. A weak activity can be noticed for compound **1**, its enantiomer **1′**, and their equimolar mixture **1″** Interestingly, for two cancer lines racemate exhibited the highest activity among these samples, although the effect was still unsatisfactory. Significantly better results were obtained for two derivatives: a phosphonate bearing big TADDOL—derived moiety **4e**, and a monocyclic diamine with trifluoromethyl substituent **6**. Compound **4e** was previously tested in our previous antiproliferative study using other cancer lines and was found as the only active compound [17]. Moreover, a mixture of epimers which was obtained from the synthesis was even more efficient in comparison with a pure enantiomer. The range of applications of compound is wider: in another study, it appeared useful for the treatment and prevention of herpes by inhibition of replication of HSV 1 virus [39]. Apparently, the TADDOL fragment combined with the rigid bicyclic imine skeleton results in high biological activity of this compound. 

A similar activity in the current study was observed for a lactam containing trifluoromethyl group **6**. This piperazine-based compound differs from the rest of the investigated compounds that bear a bicyclic skeleton. While NH, OH and C=O fragments offer numerous possibilities of interactions with biological targets by hydrogen bonds, fluorinated groups, and, in particular, CF_3_ substituent are well recognized pharmacophores [40,41,42]. Unfortunately, both derivatives were also relatively toxic for non-malignant cells, though for certain lines the selectivity was better. Since the tested compounds may interact with multiple of biological targets, e.g., hydrogen bonding, it is nearly impossible to formulate a hypotheses about their toxic effects on normal cells. Further studies on that effects should be carried out to provide insight into their mechanism of action.

## 4. Conclusions

Reaction of cyclohexanediamine with ketoesters serves as a useful route to chiral cyclic imines which can be converted to a variety of derivatives, including aminophosphonates. Here, checked also the possible use of other diamines in a similar transformation, and found that in case of ethylenediamine the course of the reaction is strongly dependent on the structure of the second reactant. This led to preparation of interesting amides, including a chiral bicyclic dilactam **10**. Another product bearing CF_3_ substituent exhibited a significant cytotoxic activity. 

We examined the effect of the prepared compounds on a variety of malignant cell lines, including HCC, MB and GBM. HUVEC, a non-malignant primary cells were used as a control. Our results indicate that 50 µM concentration of compounds **4e** and **6** decrease cell viability by more than 50% in all cell lines tested. Unfortunately, similar compound toxicity was observed in nonmalignant HUVEC cells. Further efforts to improve compound selectivity towards cancer cells are required. 

## Figures and Tables

**Table 1:** Synthesis of tricyclic dilactam **10**.

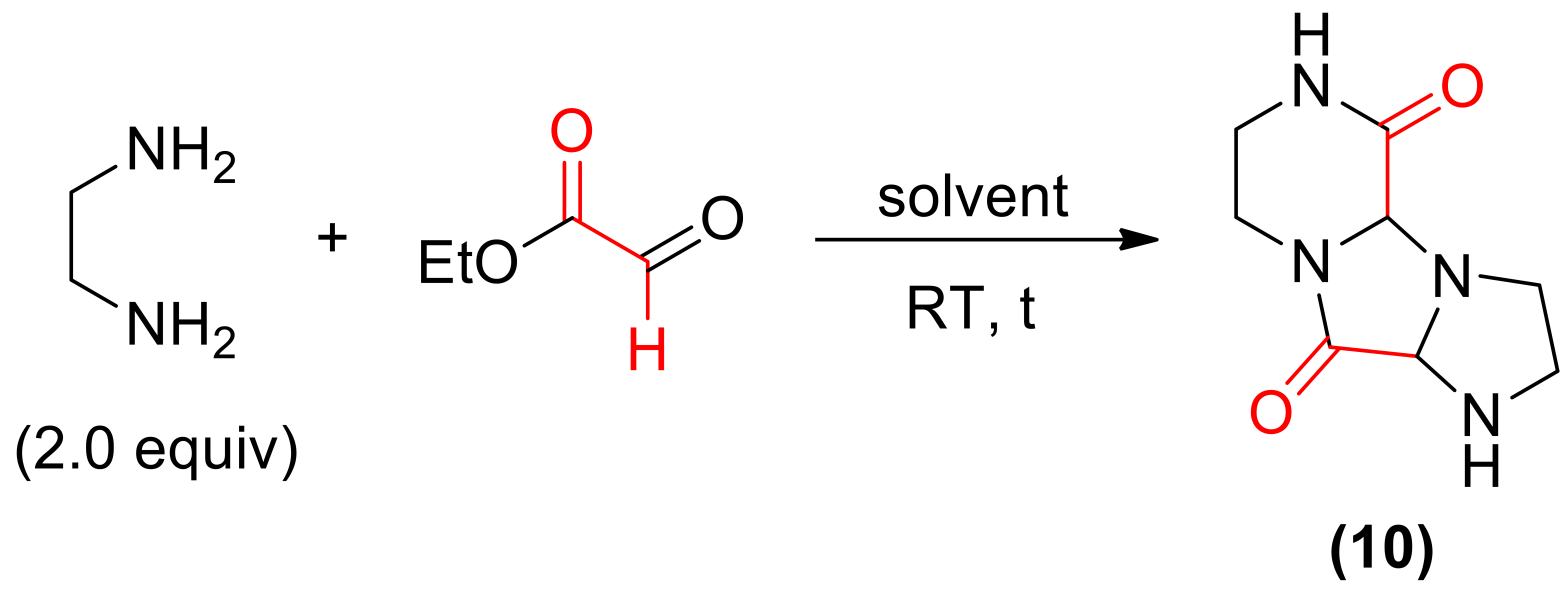				
Entry	Ethyl Glyoxylate	Solvent	T, *h*	Yield, *%*
**1**	obtained *in situ*	Et_2_O	48	24
**2**	obtained before synthesis	EtOH	48	34
**3**	polymeric form	EtOH	48	44
**4**	polymeric form	EtOH	168	67
**5**	polymeric form	EtOH	720	79

**Table 2:** TD_50_ values (in µM) of selected compounds tested in malignant and nonmalignant human cells by neutral red method.

Compounds	Hepatocellular Carcinoma Cell Lines		Medulloblastoma Cell Lines			Glioblastoma Cell	HUVEC Primary Cells
	HUH7	AKH12	DAOY	UW228-2	D283	U251	
**4e**	31.4 ± 1.2	36.6 ± 1.9	12.6 ± 1.0	30.7 ± 2.1	32 ± 1.1	12.7 ± 1.6	5.2 ± 0.9
**6**	19.4 ± 5.2	32.0 ± 1.1	16 ± 1.0	26.8 ± 1.4	38.4 ± 2.0	35.0 ± 2.7	18.7 ± 1.5

**Table 3:** TD_50_ values (in µM) of selected compounds tested in malignant and nonmalignant human cells by MTT method.

Compounds	Medulloblastoma Cell Line D425	HUVEC Primary Cells
**4e**	5.9 ± 0.1	7.2 ± 1.4
**6**	28.3 ± 0.9	24.0 ± 2.3

## Data Availability

Not applicable.

## Supplementary Materials

The following are available online at https://www.mdpi.com/article/10.3390/ma14092138/s1, Figure S1: (a) ^1^H NMR and (b) ^13^C NMR spectra of 4-trifluoromethyl-(1*R*,6*R*)-3-oxo-2,5-diazabicyclo[4.4.0]dec-4-ene (**2b**), Figure S2: (a) ^1^H NMR and (b) ^13^C NMR spectra of 3-hydroxy-3-(trifluoromethyl)piperazin-2-one (**6**), Figure S3: (a) ^1^H NMR and (b) ^13^C NMR spectra of *N*,*N*’-(ethane-1,2-diyl)bis(2-oxo-2-phenylacetamide) (**8**), Figure S4: (a) ^1^H NMR, (b) ^13^C NMR and (c) DEPT-135 spectra of 3,8-diphenyl-1,4,7,10-tetraazacyclododeca-3,7-diene-2,9-dione (**9**), Figure S5: (a) ^1^H NMR and (b) ^13^C NMR spectra of hexahydroimidazo[1′,2′:3,4]imidazo[1,2-a]pyrazine-5,10(4aH,6H)-dione (**10**), Figure S6: X-ray structure of hexahydroimidazo[1′,2′:3,4]imidazo[1,2-a]pyrazine-5,10(4a*H*,6*H*)-dione (10), Figure S7: Effect of investigated compounds on HUH7 cell line, Figure S8: Impact of aminophosphonates **4e** and **6** on HUH7 in contrast to HUVEC, Figure S9: Viability and cytotoxicity effect of investigated compounds on AKH12 cells, Figure S10: Impact of aminophosphonates 4e and 6 on AKH12 compared to HUVEC, Figure S11: Influence of investigated compounds on DAOY cell viability and cytotoxicity, Figure S12: Sensitivity of DAOY to 4e and 6 in contrast to HUVEC cell lines, Figure S13: Effect of studied compounds on viability of UW228-2 cell line, Figure S14: Impact of compounds **4e** and **6** on UW228-2 in contrast to HUVEC, Figure S15: Cytotoxicity of studied compounds on D425 cells, Figure S16: Effect of aminophosphonates **4e** and **6** on viability of D425 in comparison with HUVEC cell lines, Figure S17: Influence of studied compounds treatment on D283 cell viability and cytotoxicity, Figure S18: Efficacy of aminophosphonates on cell viability of D283 compared to HUVEC cell lines, Figure S19: The effect of studied compounds on the viability of U251 cells, Figure S20: Efficacy of aminophosphonates **4e** and **6** on cell viability of U251 compared to HUVEC cell lines. Table S1: Crystal data and structure refinement for *N*,*N*′-(ethane-1,2-diyl)bis(2-oxo-2-phenylacetamide) (**8**), Table S2: Atomic coordinates (×104) and equivalent isotropic displacement parameters (Å^2^ × 10^3^) for *N*,*N*′-(ethane-1,2-diyl)bis(2-oxo-2-phenylacetamide) (**8**), Table S3: Bond lengths [Å] and angles [°] for *N*,*N*′-(ethane-1,2-diyl)bis(2-oxo-2-phenylacetamide) (**8**), Table S4: Anisotropic displacement parameters (Å^2^ × 10^3^) for *N*,*N*′-(ethane-1,2-diyl)bis(2-oxo-2-phenylacetamide) (**8**), Table S5: Hydrogen coordinates (×10^4^) and isotropic displacement parameters (Å2 × 10 3) for *N*,*N*′-(ethane-1,2-diyl)bis(2-oxo-2-phenylacetamide) (**8**), Table S6: Torsion angles [°] for *N*,*N*′-(ethane-1,2-diyl)bis(2-oxo-2-phenylacetamide) (**8**), Table S7: Hydrogen bonds for *N*,*N*′-(ethane-1,2-diyl)bis(2-oxo-2-phenylacetamide) (**8**) [Å and °], Table S8: Crystal data and structure refinement for 3,8-Diphenyl-1,4,7,10-tetraazacyclododeca-3,7-diene-2,9-dione (**9**), Table S9: Atomic coordinates (×10^4^) and equivalent isotropic displacement parameters (Å^2^ × 10^3^) for 3,8-Diphenyl-1,4,7,10-tetraazacyclododeca-3,7-diene-2,9-dione (**9**), Table S10: Bond lengths [Å] and angles [°] for 3,8-Diphenyl-1,4,7,10-tetraazacyclododeca-3,7-diene-2,9-dione (**9**), Table S11: Anisotropic displacement parameters (Å^2^ × 10^3^) for 3,8-Diphenyl-1,4,7,10-tetraazacyclododeca-3,7-diene-2,9-dione (**9**), Table S12: Hydrogen coordinates (×10^4^) and isotropic displacement parameters (Å^2^ × 10 ^3^) for 3,8-Diphenyl-1,4,7,10-tetraazacyclododeca-3,7-diene-2,9-dione (**9**), Table S13: Torsion angles [°] for 3,8-Diphenyl-1,4,7,10-tetraazacyclododeca-3,7-diene-2,9-dione (**9**), Table S14: Hydrogen bonds for 3,8-Diphenyl-1,4,7,10-tetraazacyclododeca-3,7-diene-2,9-dione (**9**), Figure S6: X-ray structure of hexahydroimidazo[1′,2′:3,4]imidazo[1,2-*a*]pyrazine-5,10(4a*H*,6*H*-dione (**10**), Table S15: Crystal data and structure refinement for hexahydroimidazo[1′,2′:3,4]imidazo[1,2-*a*]pyrazine-5,10(4a*H*,6*H*-dione (**10**), Table S16: Atomic coordinates (×10^4^) and equivalent isotropic displacement parameters (Å^2^ × 10^3^) for hexahydroimidazo[1′,2′:3,4]imidazo[1,2-*a*]pyrazine-5,10(4a*H*,6*H*)-dione (**10**), Table S17: Bond lengths [Å] and angles [°] for hexahydroimidazo[1′,2′:3,4]imidazo[1,2-*a*]pyrazine-5,10(4a*H*,6*H*)-dione (**10**), Table S18: Anisotropic displacement parameters (Å^2^ × 10^3^) for hexahydroimidazo[1′,2′:3,4]imidazo[1,2-*a*]pyrazine-5,10(4a*H*,6*H*)-dione (**10**), Table S19: Hydrogen coordinates (×10^4^) and isotropic displacement parameters (Å^2^ × 10 ^3^) for hexahydroimidazo[1′,2′:3,4]imidazo[1,2-*a*]pyrazine-5,10(4a*H*,6*H*)-dione (**10**), Table S20: Torsion angles [°] for hexahydroimidazo[1′,2′:3,4]imidazo[1,2-*a*]pyrazine-5,10(4a*H*,6*H*)-dione (**10**), Table S21: Hydrogen bonds for hexahydroimidazo[1′,2′:3,4]imidazo[1,2-*a*]pyrazine-5,10(4a*H*,6*H*)-dione (**10**).
