# Supplementary material for: Cytotoxic Activity of Piperazin-2-One-Based Structures: Cyclic Imines, Lactams, Aminophosphonates, and Their Derivatives
Source: Materials (Basel). 2021 Apr 22;14(9):2138. doi: 10.3390/ma14092138 (PMC8122789; doi:10.3390/ma14092138)
Supplement: Supplementary file 1 [file materials-14-02138-s001.zip › materials-1172118-supplementary.pdf]

Supporting Information

Cytotoxic Activity of Piperazin-2-One-Based Structures: Cyclic Imines, Lactams, Aminophosphonates, and Their Derivatives

Contents

Experimental procedures 3

Spectra 4

Figure S1. (a) ^{1}H NMR and (b) ^{13}C NMR spectra of 4-trifluoromethyl-(1*R*,6*R*)-3-oxo-2,5-diazabicyclo[4.4.0]dec-4-ene (**2b**) 4

Figure S2. (a) ^{1}H NMR and (b) ^{13}C NMR spectra of 3-hydroxy-3-(trifluoromethyl)piperazin-2-one (**6**) 5

Figure S3. (a) ^{1}H NMR and (b) ^{13}C NMR spectra of *N*,*N*'-(ethane-1,2-diyl)bis(2-oxo-2-phenylacetamide) (8). 6

Figure S4. (a) ^{1}H NMR, (b) ^{13}C NMR and (c) DEPT-135 spectra of 3,8-diphenyl-1,4,7,10-tetraazacyclododeca-3,7-diene-2,9-dione (9). 8

Figure S5. (a) ^{1}H NMR and (b) ^{13}C NMR spectra of hexahydroimidazo[1',2':3,4]imidazo[1,2-a]pyrazine-5,10(4aH,6H)-dione (10). 9

Crystallographic data 10

X-ray structure of *N*,*N*'-(ethane-1,2-diyl)bis(2-oxo-2-phenylacetamide) (8) 10

Table S1. Crystal data and structure refinement for *N*,*N*'-(ethane-1,2-diyl)bis(2-oxo-2-phenylacetamide) (8). 10

Table S2. Atomic coordinates (x 10^4) and equivalent isotropic displacement parameters ($Å^2$x 10^3) for *N*,*N*'-(ethane-1,2-diyl)bis(2-oxo-2-phenylacetamide) (8). 11

Table S3. Bond lengths [Å] and angles [°] for *N*,*N*'-(ethane-1,2-diyl)bis(2-oxo-2-phenylacetamide) (8). 12

Table S4. Anisotropic displacement parameters ($Å^2$x 10^3) for *N*,*N*'-(ethane-1,2-diyl)bis(2-oxo-2-phenylacetamide) (8). 14

Table S5. Hydrogen coordinates (x 10^4) and isotropic displacement parameters ($Å^2$x 10^3) for *N*,*N*'-(ethane-1,2-diyl)bis(2-oxo-2-phenylacetamide) (8). 14

Table S6. Torsion angles [°] for *N*,*N*'-(ethane-1,2-diyl)bis(2-oxo-2-phenylacetamide) (8). 15

Table S7. Hydrogen bonds for *N*,*N*'-(ethane-1,2-diyl)bis(2-oxo-2-phenylacetamide) (8) [Å and °].15

X-ray structure of 3,8-Diphenyl-1,4,7,10-tetraazacyclododeca-3,7-diene-2,9-dione (9) 16

Table S8. Crystal data and structure refinement for 3,8-Diphenyl-1,4,7,10-tetraazacyclododeca-3,7-diene-2,9-dione (9) 16

Table S9. Atomic coordinates (x 10^4) and equivalent isotropic displacement parameters ($Å^2$x 10^3) for 3,8-Diphenyl-1,4,7,10-tetraazacyclododeca-3,7-diene-2,9-dione (9) 17

Table S10. Bond lengths [Å] and angles [°] for 3,8-Diphenyl-1,4,7,10-tetraazacyclododeca-3,7-diene-2,9-dione (9). 18

Table S11. Anisotropic displacement parameters ($Å^2$x 10^3) for 3,8-Diphenyl-1,4,7,10-tetraazacyclododeca-3,7-diene-2,9-dione (9) 22

Table S12. Hydrogen coordinates (x 104) and isotropic displacement parameters (Å2x 10 3) for 3,8-Diphenyl-1,4,7,10-tetraazacyclododeca-3,7-diene-2,9-dione (9). 23

Table S13. Torsion angles [°] for 3,8-Diphenyl-1,4,7,10-tetraazacyclododeca-3,7-diene-2,9-dione (9).24

Table S14. Hydrogen bonds for 3,8-Diphenyl-1,4,7,10-tetraazacyclododeca-3,7-diene-2,9-dione (9). 25

X-ray structure of hexahydroimidazo[1',2':3,4]imidazo[1,2-*a*]pyrazine-5,10(4a*H*,6*H*)-dione (10).......26

Figure S6. X-ray structure of hexahydroimidazo[1',2':3,4]imidazo[1,2-*a*]pyrazine-5,10(4a*H*,6*H*)-dione (10) 26

Table S15. Crystal data and structure refinement for hexahydroimidazo[1',2':3,4]imidazo[1,2-*a*]pyrazine-5,10(4a*H*,6*H*)-dione (10). 26

Table S16. Atomic coordinates (x 104) and equivalent isotropic displacement parameters (Å2x 103) for hexahydroimidazo[1',2':3,4]imidazo[1,2-a]pyrazine-5,10(4aH,6H)-dione (10)...27

Table S17. Bond lengths [Å] and angles [°] for hexahydroimidazo[1',2':3,4]imidazo[1,2-a]pyrazine-5,10(4aH,6H)-dione (10). 28

Table S18. Anisotropic displacement parameters ($Å^2$x 10^3) for hexahydroimidazo[1',2':3,4]imidazo[1,2-*a*]pyrazine-5,10(4a*H*,6*H*)-dione (10). 30

Table S19. Hydrogen coordinates (x 10^4) and isotropic displacement parameters ($Å^2$x 10 ^{3}).....31

for hexahydroimidazo[1',2':3,4]imidazo[1,2-*a*]pyrazine-5,10(4a*H*,6*H*)-dione (10)............................31

Table S20. Torsion angles [°] for hexahydroimidazo[1',2':3,4]imidazo[1,2-*a*]pyrazine-5,10(4a*H*,6*H*)-dione (10). 31

Table S21. Hydrogen bonds for hexahydroimidazo[1',2':3,4]imidazo[1,2-*a*]pyrazine-5,10(4a*H*,6*H*)-dione (10) [Å and °]. 32

Evaluation of cytotoxicity 33

Figure S7. Effect of investigated compounds on HUH7 cell line. 33

Figure S8. Impact of aminophosphonates 4e and 6 on HUH7 in contrast to HUVEC. 34

Figure S9.Viability and cytotoxicity effect of investigated compounds on AKH12 cells. 35

Figure S10. Impact of aminophosphonates 4e and 6 on AKH12 compared to HUVEC. 36

Figure S11. Influence of investigated compounds on DAOY cell viability and cytotoxicity. 37

Figure S12. Sensitivity of DAOY to 4e and 6 in contrast to HUVEC cell lines. 38

Figure S13. Effect of studied compounds on viability of UW228-2 cell line.......................................39

Figure S14. Impact of compounds 4e and 6 on UW228-2 in contrast to HUVEC. 40

Figure S15. Cytotoxicity of studied compounds on D425 cells. 41

Figure S16. Effect of aminophosphonates 4e and 6 on viability of D425 in comparison with HUVEC cell lines. 42

Figure S17. Influence of studied compounds treatment on D283 cell viability and cytotoxicity. ..43

Figure S18. Efficacy of aminophosphonates on cell viability of D283 compared to HUVEC cell lines. 44

Figure S19. The effect of studied compounds on the viability of U251 cells. 45

Figure S20. Efficacy of aminophosphonates 4e and 6 on cell viability of U251 compared to HUVEC cell lines. 46

Experimental procedures

Procedure for 2b

(1*R*,2*R*)-1,2-diaminocyclohexane (2.00 mmol, 228 mg, 2.00 equiv) was dissolved in 2-PrOH (4 ml). To the stirred solution was added ethyl 3,3,3-trifluoro pyruvate (1.00 mmol, 0.133 ml, 1.00 equiv) and the mixture was stirred for 24 hours at room temperature (293 K). The precipitate was filtered off, washed with three portions of 2-PrOH (3 x 2 ml) and air-dried overnight.

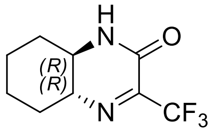

2b 4-trifluoromethyl-(1*R*,6*R*)-3-oxo-2,5-diazabicyclo[4.4.0]dec-4-ene

Colorless solid (0.183 g, 83%). Mp. 184-185 °C. $[\alpha]_D^{20}$ −40 (c 0.90, acetone). ^{1}H NMR (400 MHz, $(CD_3)_2DO$): δ 7.42 (br. s, 1H), 3.03 (ddd, *J* = 11.2, 9.4, 3.7 Hz, 1H), 2.80-2.86 (m, 1H), 1.84-1.95 (m, 2H), 1.68-1.74 (m, 2H), 1.18-1.42 (m, 4H). ^{13}C NMR (100 MHz, $(CD_3)_2DO$): δ 164.9, 123.4 (q, *J* = 285.6), 82.7 (q, *J* = 28.9), 57.3, 53.5, 30.0, 29.8, 24.0, 23.6. IR (KBr): 3351, 3211, 3108, 2949, 2888, 2869, 1679, 1652, 1477, 1427, 1192, 1155, 1061, 1000, 806 cm^{-1}. HRMS (ESI+, m/z): calcd. for $C_9H_{12}F_3N_2O$ ([M + H]$^+$), 221.0902, found, 221.0910.

Procedure for 9

Ethylenediamine (10.0 mmol, 601 mg, 0.668 mL, 1.00 equiv) was dissolved in 2-PrOH (20 ml). To the stirred solution was added ethyl 2-oxo-2-phenylacetate (10.0 mmol, 1.78 g, 1.00 equiv) and the mixture was stirred for 48 hours at room temperature (293 K). The solvent was removed under vacuo and the products were isolated by silica gel column chromatography (eluent: CH_2Cl_2/MeOH 80:20 v/v).

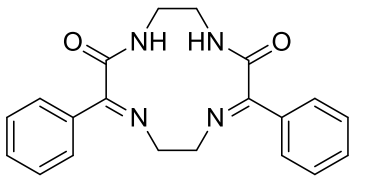

9 3,8-diphenyl-1,4,7,10-tetraazacyclododeca-3,7-diene-2,9-dione

Colorless solid (0.045 g, 13%). Mp. 291-293 °C. ^{1}H NMR (600 MHz, DMSO-d_6): δ 8.70 (br. s, 2H), 7.72-7.74 (m, 4H), 7.46-7.52 (m, 6H), 4.08 (br. s, 4H), 3.40 (br. s, 2H), 3.03 (br. s, 2H). ^{13}C NMR (151 MHz, DMSO-d_6): δ 167.3 (2C overlapped), 165.9 (2C overlapped), 135.1 (2C overlapped), 131.3 (2C overlapped), 129.0 (4C overlapped), 127.7 (4C overlapped), 55.6 (2C overlapped), 37.2 (2C overlapped). HRMS (ESI+, *m*/*z*): calcd. for $C_{20}H_{21}N_4O_2$ ([M + H]$^+$), 349.1664, found, 349.1659.

Procedure for 10

Ethylenediamine (0.668 mL, 10.0 mmol) was dissolved in ethanol (20 ml). To the stirred solution ethyl glyoxalate (~50% in toluene, 2.10 ml, 10.0 mmol, 1.00 equiv) was added and the mixture was stirred for 48 hours at room temperature (293 K). The solvent was removed under vacuo and the product were isolated by silica gel column chromatography (eluent: CH_2Cl_2/MeOH 80:20 v/v).

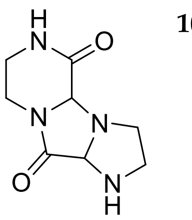

10 hexahydroimidazo[1',2':3,4]imidazo[1,2-a]pyrazine-5,10(4aH,6H)-dione

Colorless solid (0.153 g, 78%). Mp. 185-187 °C. ^{1}H NMR (500 MHz, D_2O): δ 4.68 (d, *J* = 1.5 Hz, 1H), 4.44 (d, *J* = 1.0 Hz, 1H), 3.73-3.81 (m, 1H), 3.24-3.37 (m, 3H), 3.09 (ddd, *J* = 12.5, 5.5, 1.0 Hz, 1H), 3.01 (ddd, *J* = 12.2, 6.0, 1.5 Hz, 1H), 2.87 (dq, *J* = 12.0, 5.5 Hz, 1H) 2.43 (dq, *J* = 11.5, 6.0 Hz, 1H). ^{13}C NMR (100 MHz, DMSO-d_6): δ 169.5, 168.5, 79.4, 75.9, 55.4, 43.8, 39.9, 37.2. IR (KBr): 3433, 3319, 3224, 2933, 2882, 1690, 1667, 1473, 1328, 1137, 1084, 975, 806 cm^{-1}. HRMS (ESI+, *m*/*z*): Found, 197.1032; calcd. for $C_8H_{13}N_4O_2$ ([M + H]$^+$), 197.1039.

Spectra

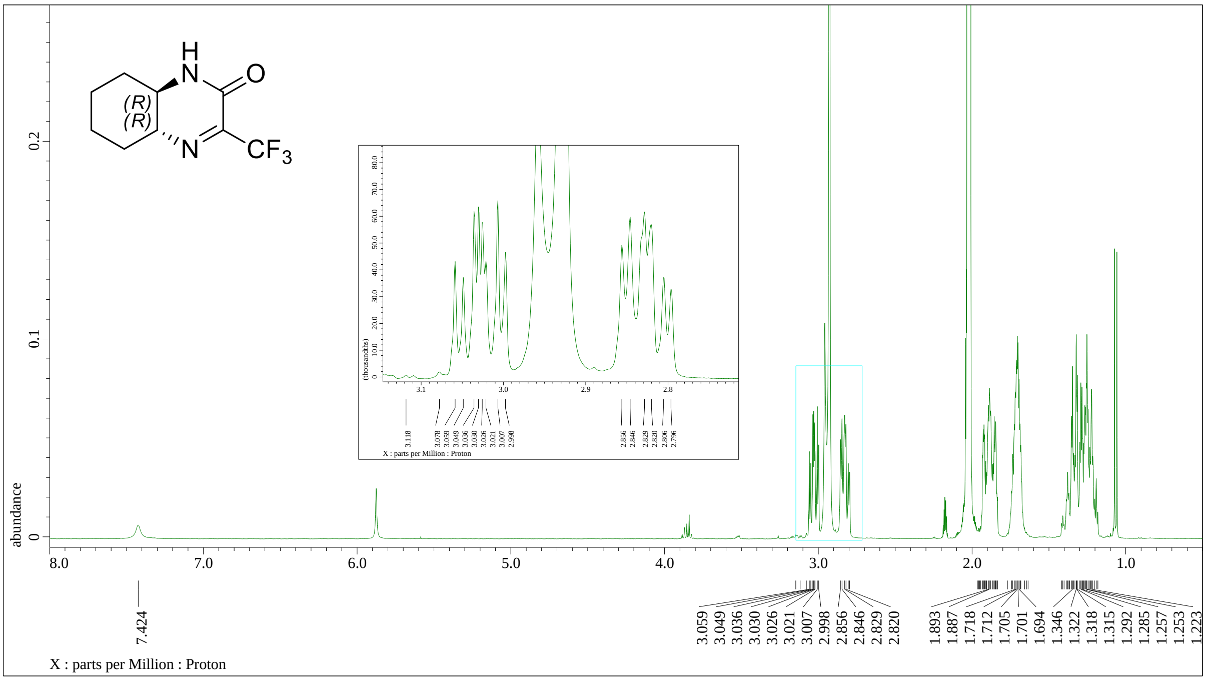

(**a**)

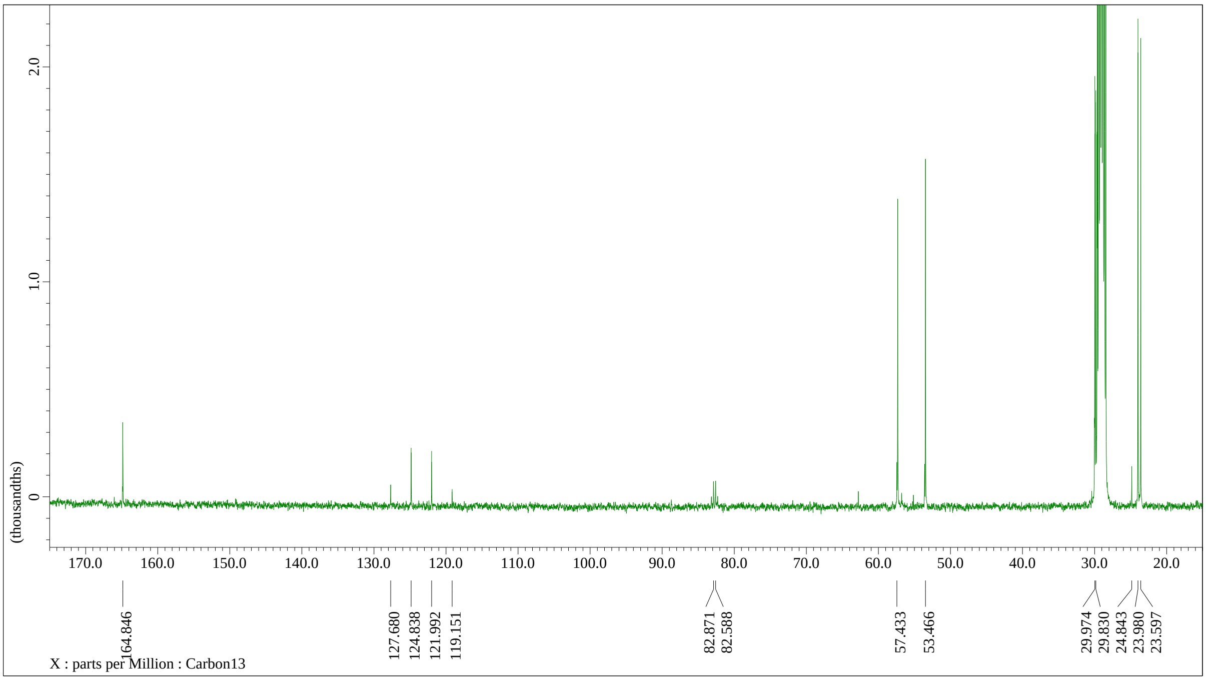

(**b**)

Figure S1. (**a**) ^{1}H NMR and (**b**) ^{13}C NMR spectra of 4-trifluoromethyl-(1*R*,6*R*)-3-oxo-2,5-diazabicyclo[4.4.0]dec-4-ene (**2b**).

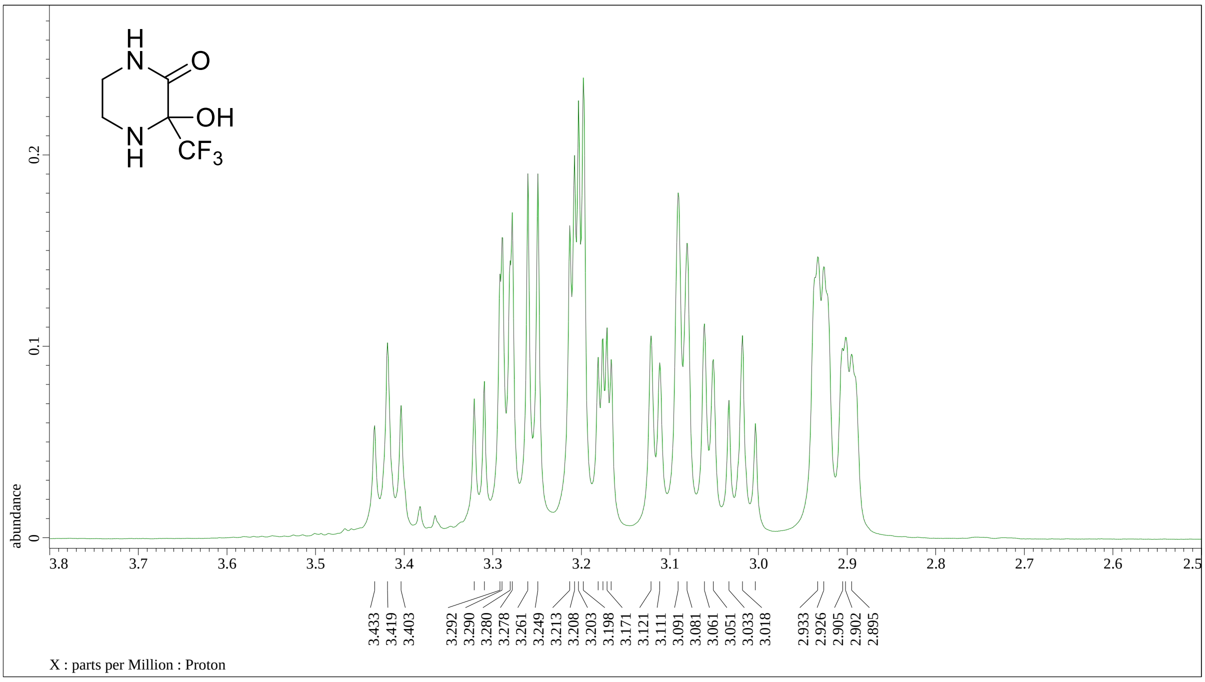

(a)

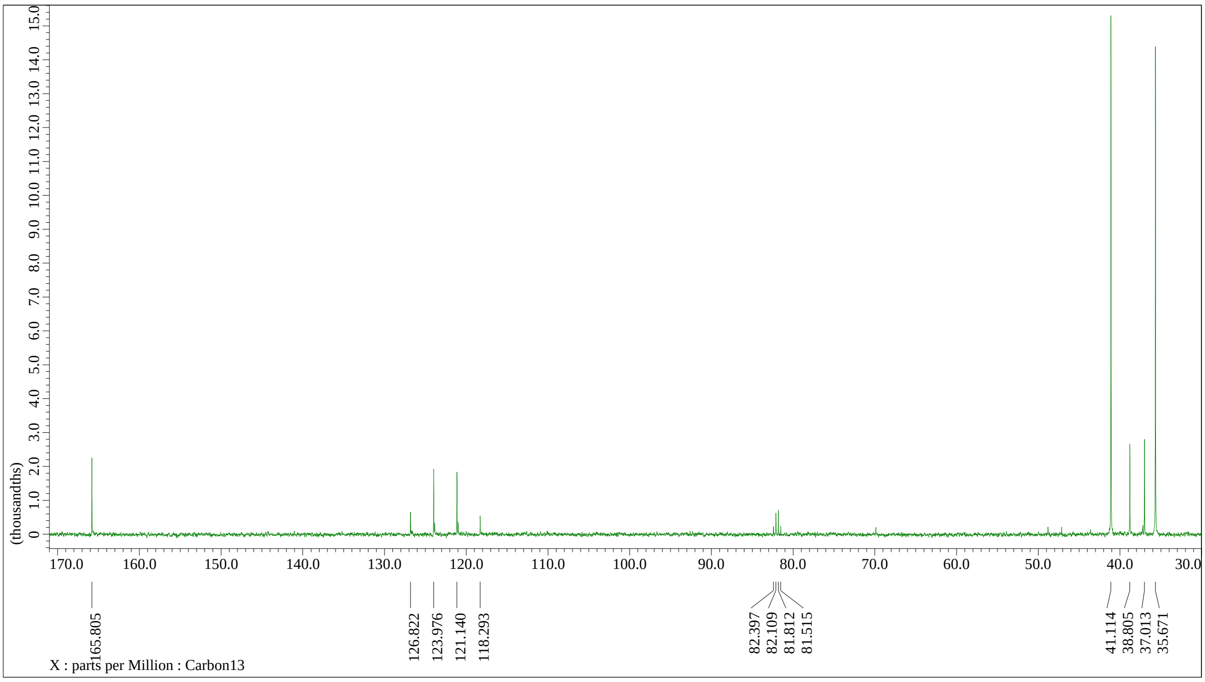

(b)

Figure S2. (**a**) ^{1}H NMR and (**b**) ^{13}C NMR spectra of 3-hydroxy-3-(trifluoromethyl)piperazin-2-one (**6**).

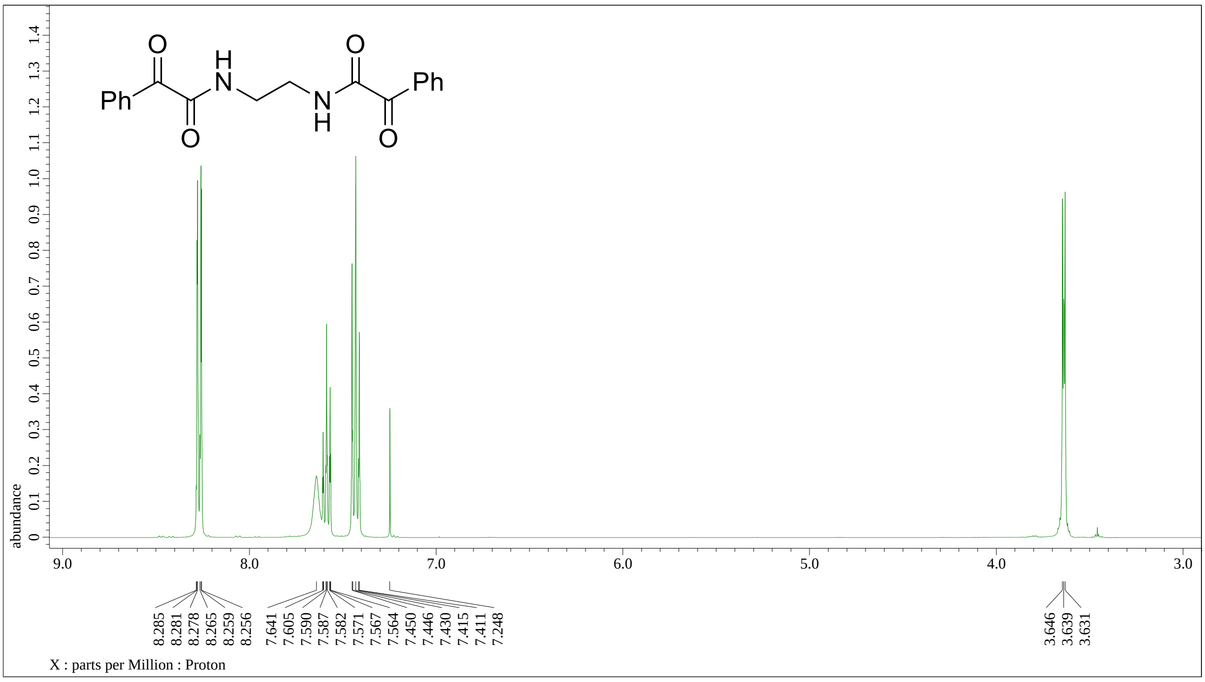

(a)

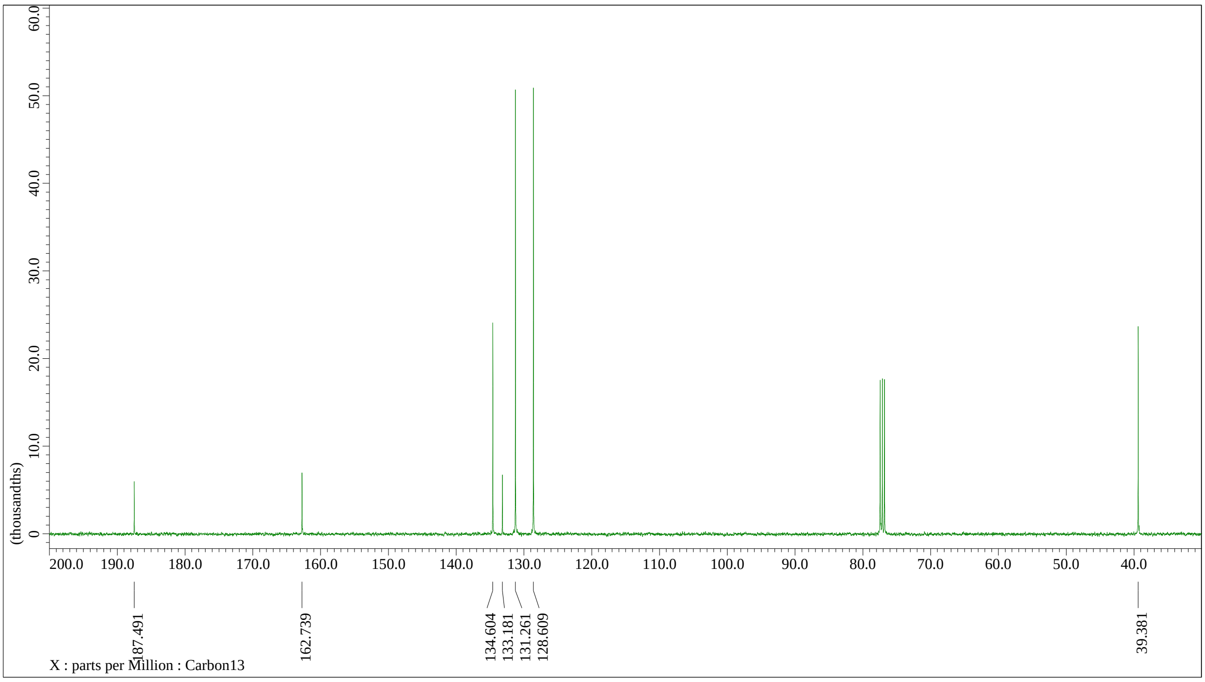

(b)

Figure S3. (**a**) ^{1}H NMR and (**b**) ^{13}C NMR spectra of *N*,*N*'-(ethane-1,2-diyl)bis(2-oxo-2-phenylacetamide) (**8**).

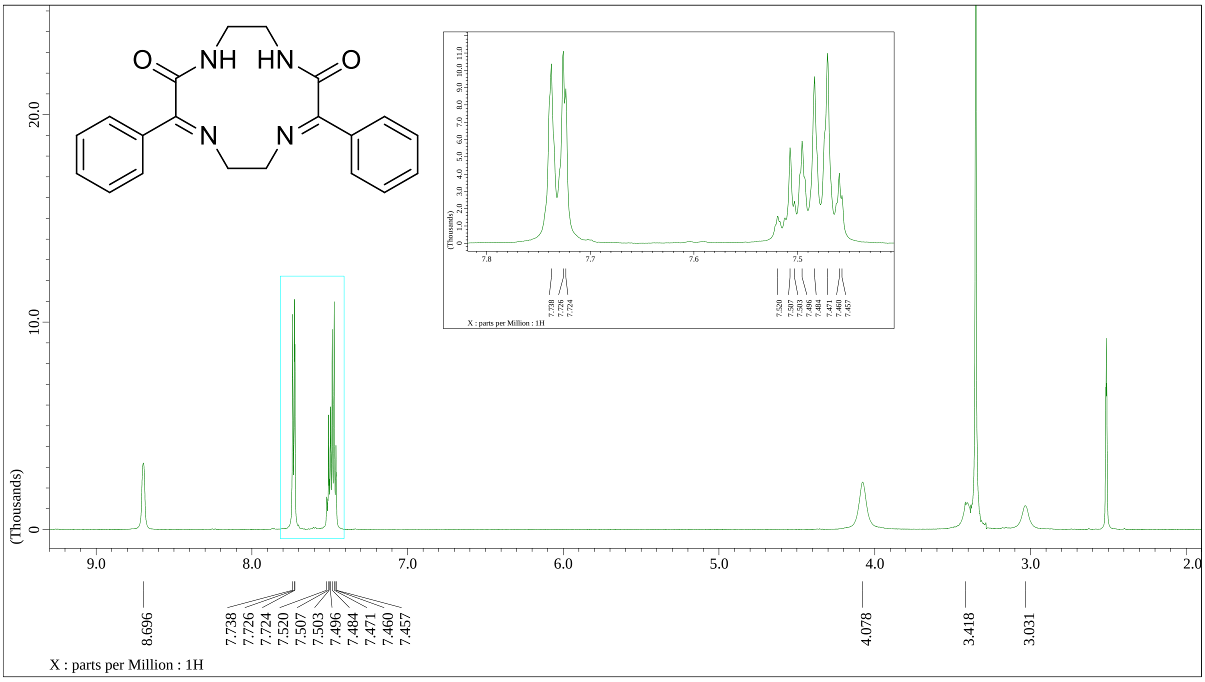
X : parts per Million : 1H
(Thousands)
8.696
7.738
7.726
7.724
7.520
7.507
7.503
7.496
7.484
7.471
7.460
7.457
4.078
3.418
3.031

(a)

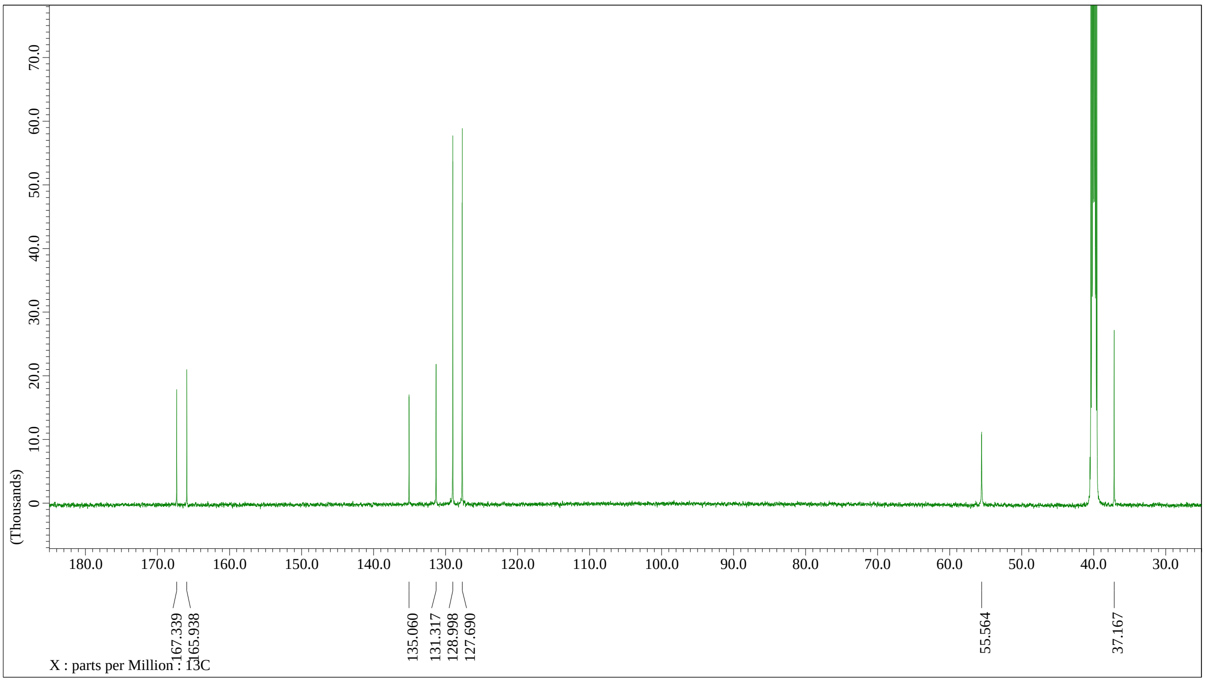
X : parts per Million : 13C
(Thousands)
167.339
165.938
135.060
131.317
128.998
127.690
55.564
37.167

(b)

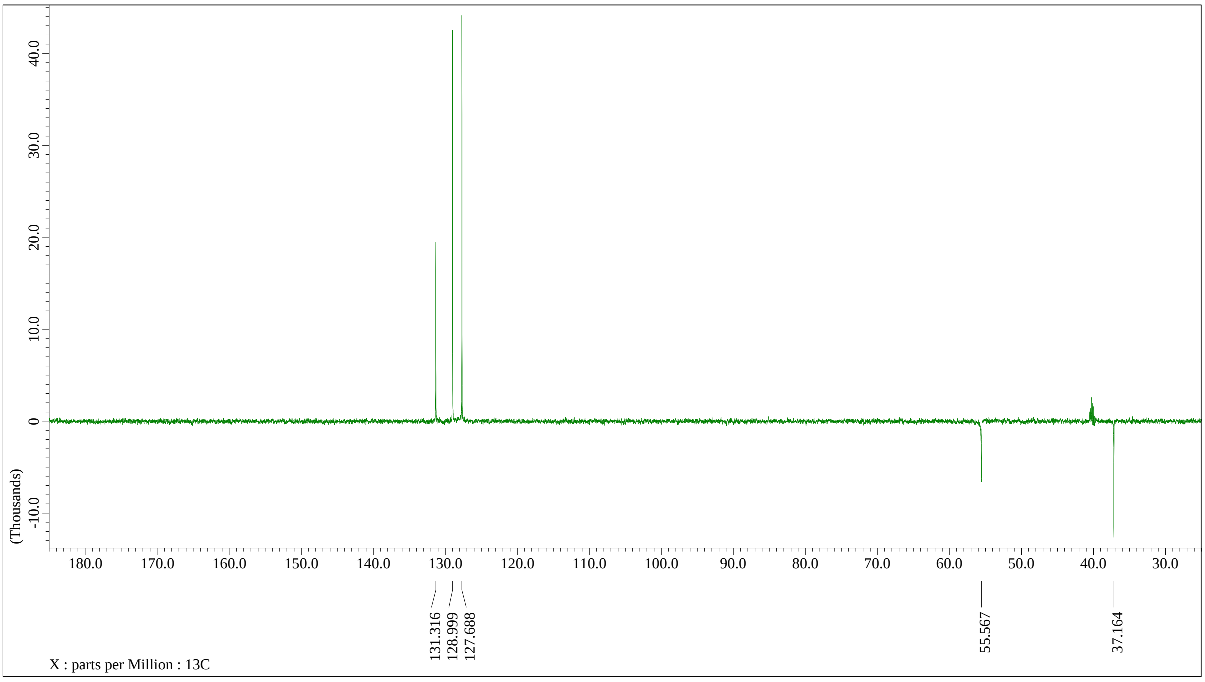

(c)

Figure S4. (**a**) ^{1}H NMR, (**b**) ^{13}C NMR and (**c**) DEPT-135 spectra of 3,8-diphenyl-1,4,7,10-tetraazacyclododeca-3,7-diene-2,9-dione (**9**).

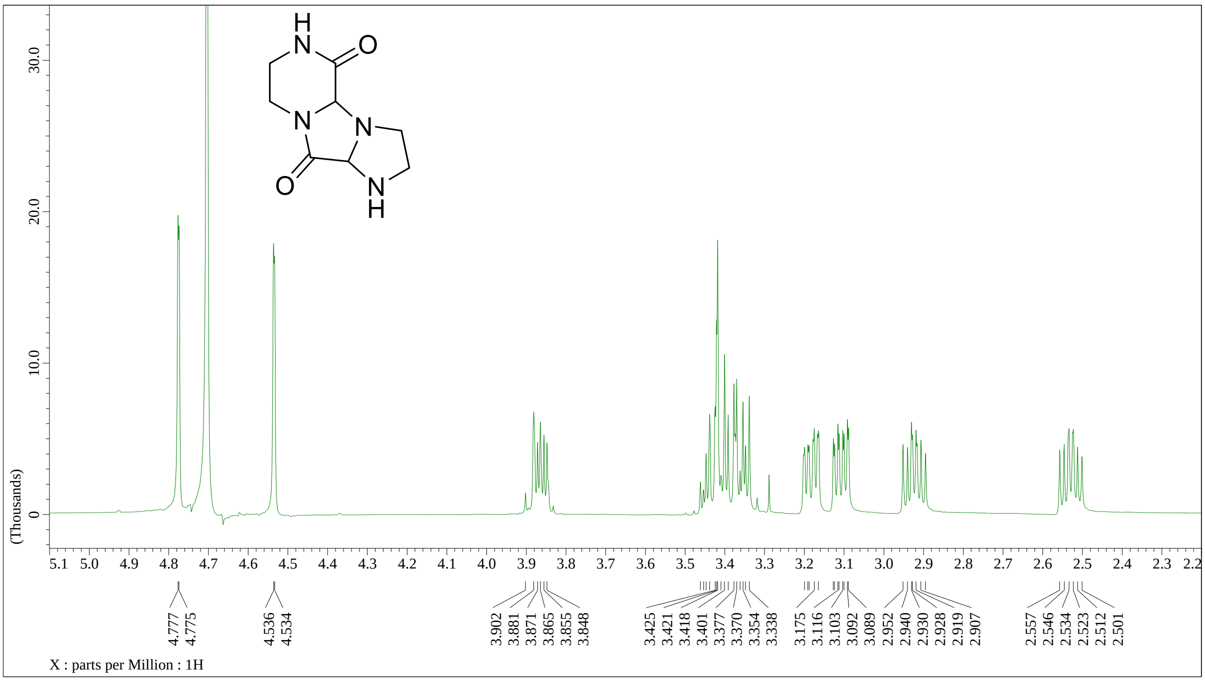

(a)

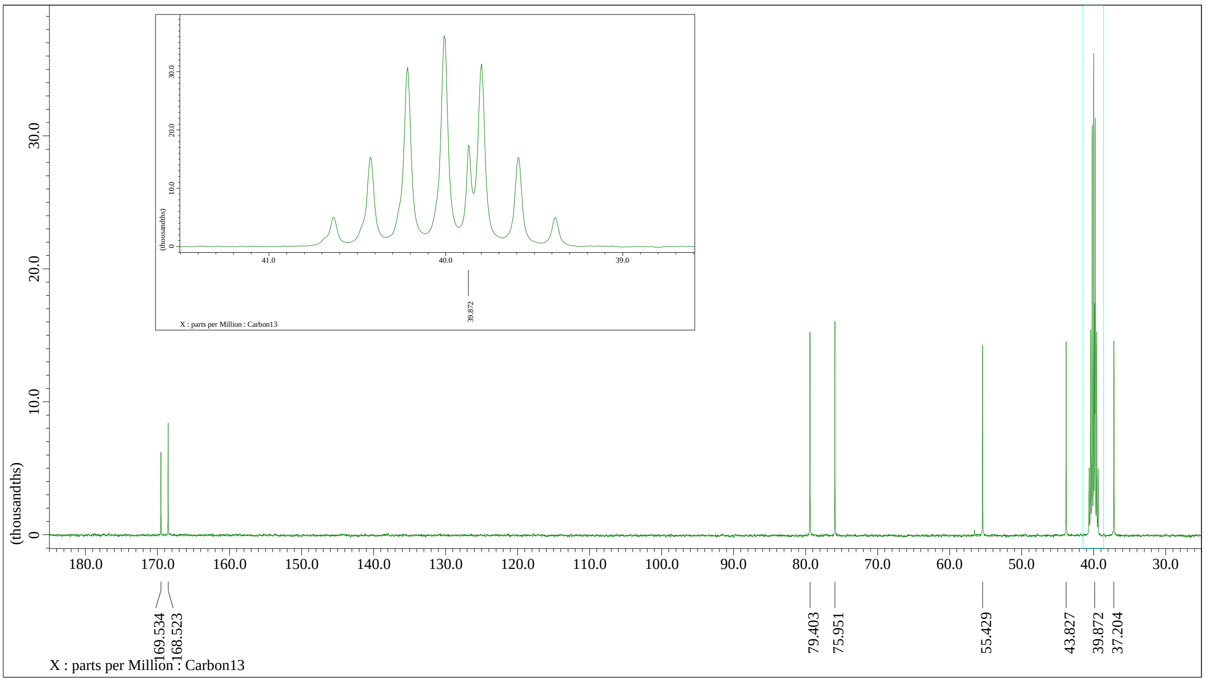

(b)

Figure S5. (**a**) ^{1}H NMR and (**b**) ^{13}C NMR spectra of hexahydroimidazo[1',2':3,4]imidazo[1,2-a]pyrazine-5,10(4aH,6H)-dione (**10**).

Crystallographic data

X-ray structure of *N*,*N*'-(ethane-1,2-diyl)bis(2-oxo-2-phenylacetamide) (8)

Table S1. Crystal data and structure refinement for *N*,*N*'-(ethane-1,2-diyl)bis(2-oxo-2-phenylacetamide) (8).

Identification code	ewokak9	
Empirical formula	C_{18} H_{16} N_2 O_4	
Formula weight	324.33	
Temperature	100(2) K	
Wavelength	0.71073 Å	
Crystal system	Triclinic	
Space group	P-1	
Unit cell dimensions	a = 5.087(3) Å	= 85.29(3)°
	b = 5.425(3) Å	= 80.17(2)°
	c = 14.429(4) Å	= 79.89(3)°
Volume	385.7(3) $Å^3$	
Z	1	
Density (calculated)	1.396 Mg/m^3	
Absorption coefficient	0.100 mm^{-1}	
F(000)	170	
Crystal size	0.23 x 0.21 x 0.20 mm^3	
Theta range for data collection	2.870 to 28.723°.	
Index ranges	-6<=h<=6, -7<=k<=7, -19<=l<=15	
Reflections collected	3248	
Independent reflections	1819 [R(int) = 0.0415]	
Completeness to theta = 25.000°	99.1 %	
Absorption correction	Analytical	
Max. and min. transmission	0.789 and 0.678	
Refinement method	Full-matrix least-squares on F^2	
Data / restraints / parameters	1819 / 1 / 109	
Goodness-of-fit on F^2	0.943	
Final R indices [I>2sigma(I)]	R1 = 0.0551, wR2 = 0.1407	
R indices (all data)	R1 = 0.0748, wR2 = 0.1498	
Largest diff. peak and hole	0.376 and -0.268 e.$Å^{-3}$	

Table S2. Atomic coordinates (x 10^4) and equivalent isotropic displacement parameters ($Å^2$x 10^3) for *N,N*'-(ethane-1,2-diyl)bis(2-oxo-2-phenylacetamide) (8).

U(eq) is defined as one third of the trace of the orthogonalized U^{ij} tensor.

	x	y	z	U(eq)
O(1)	4966(2)	7570(2)	6018(1)	24(1)
O(2)	8202(2)	7251(2)	7916(1)	24(1)
N(1)	9448(3)	7577(2)	5977(1)	18(1)
C(1)	6994(3)	7210(3)	6403(1)	17(1)
C(2)	6830(3)	6386(3)	7454(1)	17(1)
C(3)	4927(3)	4659(3)	7861(1)	19(1)
C(4)	3899(3)	3160(3)	7316(1)	21(1)
C(5)	2166(4)	1543(3)	7748(1)	25(1)
C(6)	1460(4)	1408(4)	8717(2)	29(1)
C(7)	2479(4)	2883(4)	9265(1)	33(1)
C(8)	4204(4)	4500(4)	8839(1)	28(1)
C(9)	9960(3)	8592(3)	5012(1)	19(1)

Table S3. Bond lengths [Å] and angles [°] for *N,N'*-(ethane-1,2-diyl)bis(2-oxo-2-phenylacetamide) (8).

O(1)-C(1)	1.232(2)
O(2)-C(2)	1.216(2)
N(1)-C(1)	1.335(2)
N(1)-C(9)	1.452(2)
N(1)-H(1N)	0.90
C(1)-C(2)	1.537(2)
C(2)-C(3)	1.482(2)
C(3)-C(8)	1.395(3)
C(3)-C(4)	1.395(2)
C(4)-C(5)	1.389(2)
C(4)-H(4)	0.93
C(5)-C(6)	1.382(3)
C(5)-H(5)	0.93
C(6)-C(7)	1.387(3)
C(6)-H(6)	0.93
C(7)-C(8)	1.384(3)
C(7)-H(7)	0.93
C(8)-H(8)	0.93
C(9)-C(9)#1	1.532(3)
C(9)-H(9A)	0.97
C(9)-H(9B)	0.97
C(1)-N(1)-C(9)	122.65(14)
C(1)-N(1)-H(1N)	120.9
C(9)-N(1)-H(1N)	116.1
O(1)-C(1)-N(1)	124.45(16)
O(1)-C(1)-C(2)	121.12(14)
N(1)-C(1)-C(2)	114.38(14)
O(2)-C(2)-C(3)	123.17(15)
O(2)-C(2)-C(1)	118.36(14)
C(3)-C(2)-C(1)	118.41(14)
C(8)-C(3)-C(4)	119.26(16)
C(8)-C(3)-C(2)	117.63(15)
C(4)-C(3)-C(2)	123.10(16)
C(5)-C(4)-C(3)	120.02(17)
C(5)-C(4)-H(4)	120.0
C(3)-C(4)-H(4)	120.0
C(6)-C(5)-C(4)	120.18(17)
C(6)-C(5)-H(5)	119.9

C(4)-C(5)-H(5)	119.9
C(5)-C(6)-C(7)	120.25(17)
C(5)-C(6)-H(6)	119.9
C(7)-C(6)-H(6)	119.9
C(8)-C(7)-C(6)	119.84(19)
C(8)-C(7)-H(7)	120.1
C(6)-C(7)-H(7)	120.1
C(7)-C(8)-C(3)	120.46(18)
C(7)-C(8)-H(8)	119.8
C(3)-C(8)-H(8)	119.8
N(1)-C(9)-C(9)#1	110.71(17)
N(1)-C(9)-H(9A)	109.5
C(9)#1-C(9)-H(9A)	109.5
N(1)-C(9)-H(9B)	109.5
C(9)#1-C(9)-H(9B)	109.5
H(9A)-C(9)-H(9B)	108.1

Symmetry transformations used to generate equivalent atoms:

#1 -x+2,-y+2,-z+1

Table S4. Anisotropic displacement parameters ($Å^2$x 10^3) for *N*,*N'*-(ethane-1,2-diyl)bis(2-oxo-2-phenylacetamide) (8). The anisotropic displacement factor exponent takes the form: $-2 \quad {}^{2}[h^2 a^{*2}U^{11} + ... + 2 h k a^* b^* U^{12}]$

	U^{11}	U^{22}	U^{33}	U^{23}	U^{13}	U^{12}
O(1)	15(1)	31(1)	26(1)	4(1)	-4(1)	-6(1)
O(2)	24(1)	24(1)	26(1)	2(1)	-6(1)	-10(1)
N(1)	14(1)	21(1)	20(1)	3(1)	-2(1)	-6(1)
C(1)	16(1)	14(1)	21(1)	1(1)	-1(1)	-5(1)
C(2)	15(1)	17(1)	20(1)	1(1)	-2(1)	-2(1)
C(3)	16(1)	17(1)	23(1)	3(1)	-2(1)	-4(1)
C(4)	18(1)	17(1)	25(1)	-2(1)	0(1)	-4(1)
C(5)	22(1)	17(1)	34(1)	-3(1)	-1(1)	-6(1)
C(6)	26(1)	24(1)	35(1)	7(1)	1(1)	-11(1)
C(7)	40(1)	38(1)	23(1)	6(1)	1(1)	-18(1)
C(8)	32(1)	32(1)	25(1)	2(1)	-4(1)	-17(1)
C(9)	18(1)	20(1)	18(1)	-1(1)	1(1)	-5(1)

Table S5. Hydrogen coordinates (x 10^4) and isotropic displacement parameters ($Å^2$x 10^3) for *N*,*N'*-(ethane-1,2-diyl)bis(2-oxo-2-phenylacetamide) (8).

	x	y	z	U(eq)
H(1N)	10845	7368	6299	28
H(4)	4376	3243	6664	25
H(5)	1479	549	7384	29
H(6)	298	323	9002	34
H(7)	2004	2785	9917	40
H(8)	4887	5488	9207	34
H(9A)	8548	8313	4677	23
H(9B)	11673	7731	4697	23

Table S6. Torsion angles [°] for *N,N'*-(ethane-1,2-diyl)bis(2-oxo-2-phenylacetamide) (8).

C(9)-N(1)-C(1)-O(1)	3.6(3)
C(9)-N(1)-C(1)-C(2)	-173.84(13)
O(1)-C(1)-C(2)-O(2)	-141.80(17)
N(1)-C(1)-C(2)-O(2)	35.8(2)
O(1)-C(1)-C(2)-C(3)	35.8(2)
N(1)-C(1)-C(2)-C(3)	-146.68(15)
O(2)-C(2)-C(3)-C(8)	15.7(2)
C(1)-C(2)-C(3)-C(8)	-161.69(16)
O(2)-C(2)-C(3)-C(4)	-162.54(16)
C(1)-C(2)-C(3)-C(4)	20.0(2)
C(8)-C(3)-C(4)-C(5)	0.4(3)
C(2)-C(3)-C(4)-C(5)	178.64(15)
C(3)-C(4)-C(5)-C(6)	-0.2(3)
C(4)-C(5)-C(6)-C(7)	-0.1(3)
C(5)-C(6)-C(7)-C(8)	0.1(3)
C(6)-C(7)-C(8)-C(3)	0.0(3)
C(4)-C(3)-C(8)-C(7)	-0.3(3)
C(2)-C(3)-C(8)-C(7)	-178.65(17)
C(1)-N(1)-C(9)-C(9)#1	98.3(2)

Symmetry transformations used to generate equivalent atoms:

#1 -x+2,-y+2,-z+1

Table S7. Hydrogen bonds for *N,N'*-(ethane-1,2-diyl)bis(2-oxo-2-phenylacetamide) (8) [Å and °].

D-H...A	d(D-H)	d(H...A)	d(D...A)	<(DHA)
N(1)–H(1N)···O(1)[i]	0.90	2.09	2.817(3)	138

[i] 1+x,y,z

X-ray structure of 3,8-Diphenyl-1,4,7,10-tetraazacyclododeca-3,7-diene-2,9-dione (9)

Table S8. Crystal data and structure refinement for 3,8-Diphenyl-1,4,7,10-tetraazacyclododeca-3,7-diene-2,9-dione (9).

Identification code	ewokak49
Empirical formula	$C_{20} H_{20} N_4 O_2$
Formula weight	348.40
Temperature	90(2) K
Wavelength	0.71073 Å
Crystal system	Orthorhombic
Space group	$Pna2_1$
Unit cell dimensions	a = 27.559(3) Å
	b = 12.864(3) Å
	c = 4.914(2) Å
Volume	1742.1(8) $Å^3$
Z	4
Density (calculated)	1.328 Mg/m^3
Absorption coefficient	0.089 mm^{-1}
F(000)	736
Crystal size	0.36 x 0.13 x 0.10 mm^3
Theta range for data collection	2.725 to 28.742°.
Index ranges	-36<=h<=18, -10<=k<=17, -6<=l<=4
Reflections collected	4598
Independent reflections	2969 [R(int) = 0.0506]
Completeness to theta = 25.500°	99.4 %
Absorption correction	Analytical
Max. and min. transmission	0.956 and 0.765
Refinement method	Full-matrix least-squares on F^2
Data / restraints / parameters	2969 / 1 / 236
Goodness-of-fit on F^2	0.985
Final R indices [I>2sigma(I)]	R1 = 0.0597, wR2 = 0.0972
R indices (all data)	R1 = 0.1046, wR2 = 0.1129
Absolute structure parameter	0.0(3)
Extinction coefficient	n/a
Largest diff. peak and hole	0.259 and -0.283 e.$Å^{-3}$

Table S9. Atomic coordinates (x 10^4) and equivalent isotropic displacement parameters ($Å^2$x 10^3) for 3,8-Diphenyl-1,4,7,10-tetraazacyclododeca-3,7-diene-2,9-dione (9).

U(eq) is defined as one third of the trace of the orthogonalized U^{ij} tensor.

	x	y	z	U(eq)
O(1)	2461(1)	-430(2)	12259(5)	22(1)
O(2)	3705(1)	90(2)	2777(5)	22(1)
N(1)	3553(1)	2069(3)	6704(6)	16(1)
C(2)	3864(2)	1358(3)	6278(8)	14(1)
C(3)	3732(2)	284(3)	5225(8)	14(1)
N(4)	3639(1)	-404(3)	7206(7)	16(1)
C(5)	3444(2)	-1432(3)	6714(8)	16(1)
C(6)	2988(1)	-1622(3)	8400(8)	15(1)
N(7)	2621(1)	-838(3)	7851(6)	13(1)
C(8)	2408(2)	-277(3)	9808(8)	15(1)
C(9)	2099(2)	618(3)	8763(8)	16(1)
N(10)	2262(1)	1530(3)	8457(6)	16(1)
C(11)	2782(2)	1727(3)	8932(8)	18(1)
C(12)	3040(1)	1836(3)	6199(8)	17(1)
C(13)	4385(2)	1552(3)	6832(8)	16(1)
C(14)	4741(2)	1012(4)	5416(9)	25(1)
C(15)	5231(2)	1205(4)	5915(10)	31(1)
C(16)	5361(2)	1927(4)	7846(11)	34(1)
C(17)	5009(2)	2464(4)	9271(10)	39(1)
C(18)	4525(2)	2283(4)	8776(10)	27(1)
C(19)	1583(2)	374(3)	8143(8)	16(1)
C(20)	1342(2)	-444(4)	9394(8)	23(1)
C(121)	858(2)	-638(4)	8783(9)	28(1)
C(22)	615(2)	-34(4)	6895(9)	27(1)
C(23)	858(2)	755(4)	5594(10)	31(1)
C(24)	1336(2)	971(4)	6231(9)	27(1)

Table S10. Bond lengths [Å] and angles [°] for 3,8-Diphenyl-1,4,7,10-tetraazacyclododeca-3,7-diene-2,9-dione (9).

O(1)-C(8)	1.229(5)
O(2)-C(3)	1.231(5)
N(1)-C(2)	1.272(5)
N(1)-C(12)	1.466(5)
C(2)-C(13)	1.480(5)
C(2)-C(3)	1.519(6)
C(3)-N(4)	1.341(5)
N(4)-C(5)	1.448(5)
N(4)-H(4)	0.88
C(5)-C(6)	1.525(5)
C(5)-H(5A)	0.99
C(5)-H(5B)	0.99
C(6)-N(7)	1.454(5)
C(6)-H(6A)	0.99
C(6)-H(6B)	0.99
N(7)-C(8)	1.337(5)
N(7)-H(7)	0.88
C(8)-C(9)	1.521(6)
C(9)-N(10)	1.265(5)
C(9)-C(19)	1.489(6)
N(10)-C(11)	1.473(5)
C(11)-C(12)	1.526(6)
C(11)-H(11A)	0.99
C(11)-H(11B)	0.99
C(12)-H(12A)	0.99
C(12)-H(12B)	0.99
C(13)-C(14)	1.389(6)
C(13)-C(18)	1.395(6)
C(14)-C(15)	1.395(6)
C(14)-H(14)	0.95
C(15)-C(16)	1.375(7)
C(15)-H(15)	0.95
C(16)-C(17)	1.381(7)
C(16)-H(16)	0.95
C(17)-C(18)	1.376(6)
C(17)-H(17)	0.95
C(18)-H(18)	0.95
C(19)-C(20)	1.388(6)

C(19)-C(24)	1.391(6)
C(20)-C(121)	1.390(6)
C(20)-H(20)	0.95
C(121)-C(22)	1.383(6)
C(121)-H(21)	0.95
C(22)-C(23)	1.374(6)
C(22)-H(22)	0.95
C(23)-C(24)	1.382(6)
C(23)-H(23)	0.95
C(24)-H(24)	0.95

C(2)-N(1)-C(12)	118.4(4)
N(1)-C(2)-C(13)	120.1(4)
N(1)-C(2)-C(3)	123.2(4)
C(13)-C(2)-C(3)	116.7(4)
O(2)-C(3)-N(4)	124.3(4)
O(2)-C(3)-C(2)	122.1(4)
N(4)-C(3)-C(2)	113.5(3)
C(3)-N(4)-C(5)	123.5(3)
C(3)-N(4)-H(4)	118.2
C(5)-N(4)-H(4)	118.2
N(4)-C(5)-C(6)	111.1(3)
N(4)-C(5)-H(5A)	109.4
C(6)-C(5)-H(5A)	109.4
N(4)-C(5)-H(5B)	109.4
C(6)-C(5)-H(5B)	109.4
H(5A)-C(5)-H(5B)	108.0
N(7)-C(6)-C(5)	111.2(3)
N(7)-C(6)-H(6A)	109.4
C(5)-C(6)-H(6A)	109.4
N(7)-C(6)-H(6B)	109.4
C(5)-C(6)-H(6B)	109.4
H(6A)-C(6)-H(6B)	108.0
C(8)-N(7)-C(6)	123.1(3)
C(8)-N(7)-H(7)	118.5
C(6)-N(7)-H(7)	118.5
O(1)-C(8)-N(7)	124.5(4)
O(1)-C(8)-C(9)	121.2(4)
N(7)-C(8)-C(9)	114.3(3)

N(10)-C(9)-C(19)	120.7(4)
N(10)-C(9)-C(8)	122.9(4)
C(19)-C(9)-C(8)	116.4(4)
C(9)-N(10)-C(11)	119.1(4)
N(10)-C(11)-C(12)	109.2(3)
N(10)-C(11)-H(11A)	109.8
C(12)-C(11)-H(11A)	109.8
N(10)-C(11)-H(11B)	109.8
C(12)-C(11)-H(11B)	109.8
H(11A)-C(11)-H(11B)	108.3
N(1)-C(12)-C(11)	108.6(3)
N(1)-C(12)-H(12A)	110.0
C(11)-C(12)-H(12A)	110.0
N(1)-C(12)-H(12B)	110.0
C(11)-C(12)-H(12B)	110.0
H(12A)-C(12)-H(12B)	108.3
C(14)-C(13)-C(18)	118.9(4)
C(14)-C(13)-C(2)	120.5(4)
C(18)-C(13)-C(2)	120.6(4)
C(13)-C(14)-C(15)	120.5(4)
C(13)-C(14)-H(14)	119.8
C(15)-C(14)-H(14)	119.8
C(16)-C(15)-C(14)	119.6(5)
C(16)-C(15)-H(15)	120.2
C(14)-C(15)-H(15)	120.2
C(15)-C(16)-C(17)	120.3(5)
C(15)-C(16)-H(16)	119.9
C(17)-C(16)-H(16)	119.9
C(18)-C(17)-C(16)	120.4(5)
C(18)-C(17)-H(17)	119.8
C(16)-C(17)-H(17)	119.8
C(17)-C(18)-C(13)	120.2(5)
C(17)-C(18)-H(18)	119.9
C(13)-C(18)-H(18)	119.9
C(20)-C(19)-C(24)	118.9(4)
C(20)-C(19)-C(9)	121.8(4)
C(24)-C(19)-C(9)	119.3(4)
C(19)-C(20)-C(121)	120.0(4)
C(19)-C(20)-H(20)	120.0

C(121)-C(20)-H(20)	120.0
C(22)-C(121)-C(20)	120.5(4)
C(22)-C(121)-H(21)	119.7
C(20)-C(121)-H(21)	119.7
C(23)-C(22)-C(121)	119.4(4)
C(23)-C(22)-H(22)	120.3
C(121)-C(22)-H(22)	120.3
C(22)-C(23)-C(24)	120.5(4)
C(22)-C(23)-H(23)	119.7
C(24)-C(23)-H(23)	119.7
C(23)-C(24)-C(19)	120.6(4)
C(23)-C(24)-H(24)	119.7
C(19)-C(24)-H(24)	119.7

Symmetry transformations used to generate equivalent atoms:

Table S11. Anisotropic displacement parameters ($Å^2$x 10^3) for 3,8-Diphenyl-1,4,7,10-tetraazacyclododeca-3,7-diene-2,9-dione (9).

The anisotropic displacement factor exponent takes the form: $-2p^2[h^2 a^{*2}U^{11} + ... + 2 h k a^* b^* U^{12}]$

	U^{11}	U^{22}	U^{33}	U^{23}	U^{13}	U^{12}
O(1)	28(2)	27(2)	10(2)	2(1)	-2(1)	5(2)
O(2)	25(2)	32(2)	9(2)	-2(1)	0(1)	-4(2)
N(1)	14(2)	22(2)	12(2)	2(2)	0(2)	1(2)
C(2)	16(2)	20(2)	7(2)	5(2)	0(2)	-3(2)
C(3)	9(2)	20(2)	12(2)	3(2)	2(2)	3(2)
N(4)	18(2)	19(2)	12(2)	0(2)	-1(2)	-1(2)
C(5)	20(2)	13(2)	15(2)	0(2)	-2(2)	1(2)
C(6)	15(2)	14(2)	16(2)	1(2)	1(2)	-2(2)
N(7)	14(2)	16(2)	10(2)	2(2)	-3(2)	4(2)
C(8)	15(2)	13(2)	16(2)	2(2)	-2(2)	-5(2)
C(9)	20(3)	20(2)	8(2)	0(2)	0(2)	3(2)
N(10)	15(2)	17(2)	15(2)	-2(2)	2(2)	-1(2)
C(11)	20(2)	17(2)	17(2)	1(2)	0(2)	-1(2)
C(12)	17(2)	19(2)	16(2)	0(2)	-1(2)	3(2)
C(13)	16(2)	17(2)	15(2)	9(2)	-5(2)	-1(2)
C(14)	25(3)	24(3)	26(3)	-4(2)	2(2)	-4(2)
C(15)	14(3)	35(3)	42(3)	7(3)	5(3)	2(2)
C(16)	16(3)	34(3)	52(3)	14(3)	-14(3)	-11(2)
C(17)	34(3)	35(3)	48(3)	-7(3)	-22(3)	-5(3)
C(18)	21(3)	27(3)	34(3)	-6(2)	-8(2)	1(2)
C(19)	15(2)	16(2)	17(2)	-2(2)	1(2)	3(2)
C(20)	20(3)	24(3)	25(3)	3(2)	-3(2)	0(2)
C(121)	22(3)	31(3)	30(3)	2(2)	4(2)	-9(2)
C(22)	15(2)	32(3)	34(3)	-1(2)	-4(2)	-1(2)
C(23)	23(3)	24(3)	45(3)	5(3)	-16(3)	1(2)
C(24)	28(3)	18(2)	35(3)	8(2)	-4(2)	-3(2)

Table S12. Hydrogen coordinates (x 104) and isotropic displacement parameters (Å2x 10 3) for 3,8-Diphenyl-1,4,7,10-tetraazacyclododeca-3,7-diene-2,9-dione (9).

	x	y	z	U(eq)
H(4)	3699	-220	8898	19
H(5A)	3692	-1960	7182	19
H(5B)	3366	-1507	4757	19
H(6A)	2856	-2318	7970	18
H(6B)	3072	-1609	10358	18
H(7)	2535	-726	6151	16
H(11A)	2925	1144	9975	22
H(11B)	2823	2372	10007	22
H(12A)	3010	1182	5150	21
H(12B)	2890	2404	5128	21
H(14)	4650	508	4099	30
H(15)	5473	840	4927	37
H(16)	5694	2056	8201	41
H(17)	5102	2962	10601	47
H(18)	4285	2657	9762	33
H(20)	1507	-871	10668	27
H(21)	692	-1191	9669	33
H(22)	283	-164	6500	32
H(23)	696	1156	4247	37
H(24)	1497	1531	5355	32

Table S13. Torsion angles [°] for 3,8-Diphenyl-1,4,7,10-tetraazacyclododeca-3,7-diene-2,9-dione (9).

C(12)-N(1)-C(2)-C(13)	-178.4(3)
C(12)-N(1)-C(2)-C(3)	1.1(5)
N(1)-C(2)-C(3)-O(2)	88.0(5)
C(13)-C(2)-C(3)-O(2)	-92.5(5)
N(1)-C(2)-C(3)-N(4)	-90.6(5)
C(13)-C(2)-C(3)-N(4)	88.9(4)
O(2)-C(3)-N(4)-C(5)	-6.9(6)
C(2)-C(3)-N(4)-C(5)	171.7(4)
C(3)-N(4)-C(5)-C(6)	-126.2(4)
N(4)-C(5)-C(6)-N(7)	56.8(4)
C(5)-C(6)-N(7)-C(8)	-126.9(4)
C(6)-N(7)-C(8)-O(1)	-7.6(6)
C(6)-N(7)-C(8)-C(9)	170.3(3)
O(1)-C(8)-C(9)-N(10)	85.3(6)
N(7)-C(8)-C(9)-N(10)	-92.6(5)
O(1)-C(8)-C(9)-C(19)	-93.7(5)
N(7)-C(8)-C(9)-C(19)	88.3(5)
C(19)-C(9)-N(10)-C(11)	-176.4(3)
C(8)-C(9)-N(10)-C(11)	4.7(6)
C(9)-N(10)-C(11)-C(12)	103.7(4)
C(2)-N(1)-C(12)-C(11)	106.8(4)
N(10)-C(11)-C(12)-N(1)	178.0(3)
N(1)-C(2)-C(13)-C(14)	-153.0(4)
C(3)-C(2)-C(13)-C(14)	27.5(5)
N(1)-C(2)-C(13)-C(18)	26.7(6)
C(3)-C(2)-C(13)-C(18)	-152.8(4)
C(18)-C(13)-C(14)-C(15)	-0.6(6)
C(2)-C(13)-C(14)-C(15)	179.2(4)
C(13)-C(14)-C(15)-C(16)	0.8(7)
C(14)-C(15)-C(16)-C(17)	-0.5(7)
C(15)-C(16)-C(17)-C(18)	0.0(7)
C(16)-C(17)-C(18)-C(13)	0.2(7)
C(14)-C(13)-C(18)-C(17)	0.1(6)
C(2)-C(13)-C(18)-C(17)	-179.7(4)
N(10)-C(9)-C(19)-C(20)	-154.0(4)
C(8)-C(9)-C(19)-C(20)	25.1(5)
N(10)-C(9)-C(19)-C(24)	27.0(6)
C(8)-C(9)-C(19)-C(24)	-153.9(4)

C(24)-C(19)-C(20)-C(121)	-1.7(6)
C(9)-C(19)-C(20)-C(121)	179.3(4)
C(19)-C(20)-C(121)-C(22)	1.2(7)
C(20)-C(121)-C(22)-C(23)	0.8(7)
C(121)-C(22)-C(23)-C(24)	-2.3(7)
C(22)-C(23)-C(24)-C(19)	1.8(7)
C(20)-C(19)-C(24)-C(23)	0.2(6)
C(9)-C(19)-C(24)-C(23)	179.3(4)

Symmetry transformations used to generate equivalent atoms:

Table S14. Hydrogen bonds for 3,8-Diphenyl-1,4,7,10-tetraazacyclododeca-3,7-diene-2,9-dione (9). [Å and °].

D-H...A	d(D-H)	d(H...A)	d(D...A)	<(DHA)
N(4)–H(4)···O(2)[i]	0.88	1.95	2.816(4)	169
N(7)–H(7)···O(1)[ii]	0.88	1.96	2.832(4)	170

Symmetry codes:

[i] x,y,1+z, [ii] x,y,-1+z

X-ray structure of hexahydroimidazo[1',2':3,4]imidazo[1,2-*a*]pyrazine-5,10(4a*H*,6*H*)-dione (10)

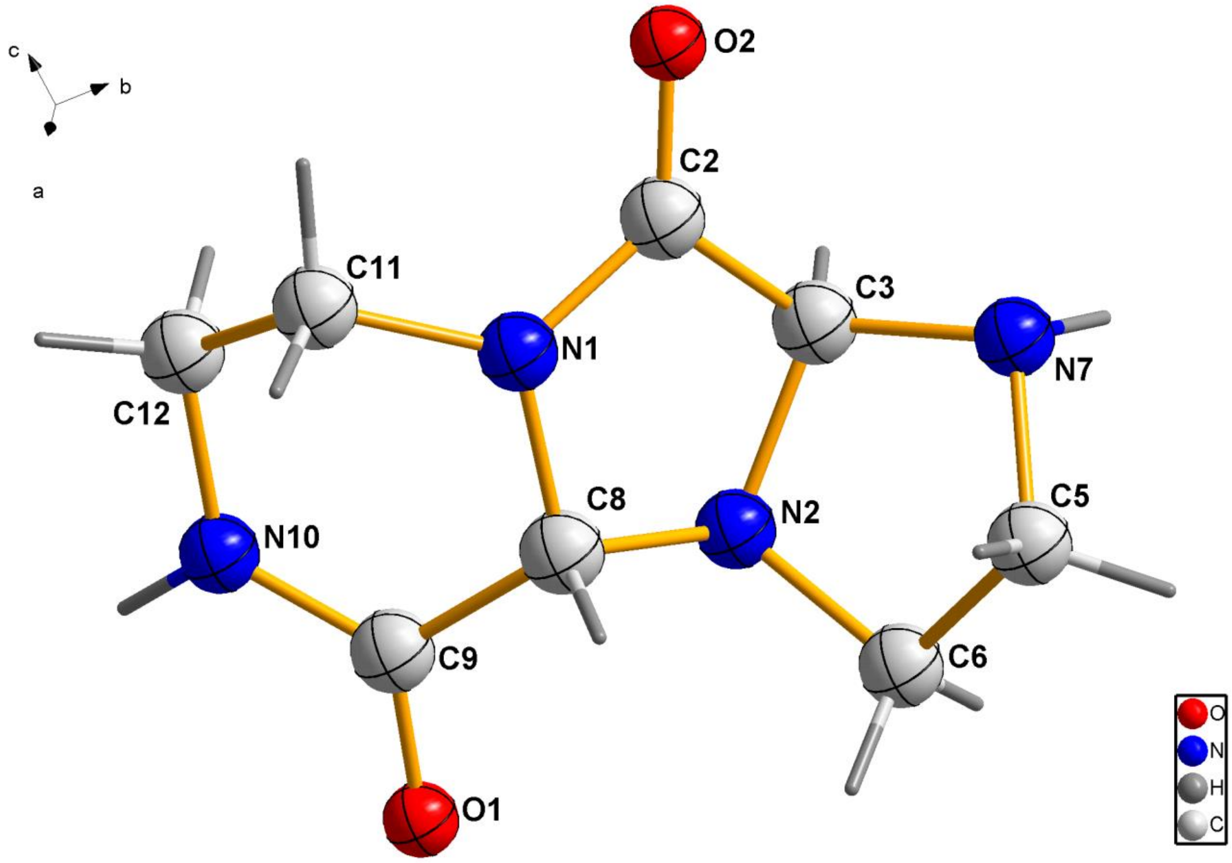

Figure S6. X-ray structure of hexahydroimidazo[1',2':3,4]imidazo[1,2-*a*]pyrazine-5,10(4a*H*,6*H*)-dione (**10**).

Table S15. Crystal data and structure refinement for hexahydroimidazo[1',2':3,4]imidazo[1,2-*a*]pyrazine-5,10(4a*H*,6*H*)-dione (10).

Identification code	ewok39	
Empirical formula	C8 H12 N4 O2	
Formula weight	196.22	
Temperature	100(2) K	
Wavelength	0.71073 Å	
Crystal system	Monoclinic	
Space group	$P2_1/c$	
Unit cell dimensions	a = 7.432(3) Å	
	b = 8.043(3) Å	= 96.21(3)°
	c = 14.462(4) Å	
Volume	859.4(5) $Å^3$	
Z	4	
Density (calculated)	1.517 Mg/m^3	
Absorption coefficient	0.113 mm^{-1}	
F(000)	416	

Crystal size	0.34 x 0.31 x 0.30 mm^3
Theta range for data collection	2.902 to 36.709°.
Index ranges	-8<=h<=10, -10<=k<=10, -23<=l<=19
Reflections collected	6331
Independent reflections	2202 [R(int) = 0.0605]
Completeness to theta = 25.000°	99.4 %
Absorption correction	Analytical
Max. and min. transmission	0.911 and 0.789
Refinement method	Full-matrix least-squares on F^2
Data / restraints / parameters	2202 / 0 / 127
Goodness-of-fit on F^2	0.934
Final R indices [I>2sigma(I)]	R1 = 0.0523, wR2 = 0.1256
R indices (all data)	R1 = 0.0739, wR2 = 0.1343
Extinction coefficient	n/a
Largest diff. peak and hole	0.330 and -0.337 e.Å^{-3}

Table S16. Atomic coordinates (x 104) and equivalent isotropic displacement parameters (Å2x 103) for hexahydroimidazo[1',2':3,4]imidazo[1,2-a]pyrazine-5,10(4aH,6H)-dione (10).

U(eq) is defined as one third of the trace of the orthogonalized U^{ij} tensor.

	x	y	z	U(eq)
O(1)	7857(2)	1505(1)	2490(1)	19(1)
O(2)	6628(2)	6315(2)	5279(1)	20(1)
N(1)	7940(2)	4282(2)	4457(1)	15(1)
N(2)	6649(2)	4798(2)	2953(1)	15(1)
N(7)	6395(2)	7722(2)	3257(1)	16(1)
N(10)	7627(2)	960(2)	4013(1)	18(1)
C(2)	6868(2)	5615(2)	4550(1)	15(1)
C(3)	5965(2)	6057(2)	3588(1)	14(1)
C(5)	7777(2)	7454(2)	2611(1)	18(1)
C(6)	7307(2)	5756(2)	2183(1)	17(1)
C(8)	8088(2)	3832(2)	3499(1)	14(1)
C(9)	7829(2)	1986(2)	3301(1)	15(1)
C(12)	7522(3)	1483(2)	4974(1)	19(1)
C(11)	8570(2)	3088(2)	5174(1)	19(1)

Table S17. Bond lengths [Å] and angles [°] for hexahydroimidazo[1',2':3,4]imidazo[1,2-a]pyrazine-5,10(4aH,6H)-dione (10).

O(1)-C(9)	1.2367(19)
O(2)-C(2)	1.2246(19)
N(1)-C(2)	1.351(2)
N(1)-C(8)	1.447(2)
N(1)-C(11)	1.453(2)
N(2)-C(8)	1.479(2)
N(2)-C(6)	1.481(2)
N(2)-C(3)	1.492(2)
N(7)-C(3)	1.469(2)
N(7)-C(5)	1.477(2)
N(7)-H(7N)	0.9018
N(10)-C(9)	1.341(2)
N(10)-C(12)	1.462(2)
N(10)-H(10N)	0.9069
C(2)-C(3)	1.520(2)
C(3)-H(3A)	1.0000
C(5)-C(6)	1.524(2)
C(5)-H(5A)	0.9900
C(5)-H(5B)	0.9900
C(6)-H(6A)	0.9900
C(6)-H(6B)	0.9900
C(8)-C(9)	1.521(2)
C(8)-H(8)	1.0000
C(12)-C(11)	1.519(2)
C(12)-H(12A)	0.9900
C(12)-H(12B)	0.9900
C(11)-H(11A)	0.9900
C(11)-H(11B)	0.9900
C(2)-N(1)-C(8)	113.61(13)
C(2)-N(1)-C(11)	126.96(14)
C(8)-N(1)-C(11)	117.75(13)
C(8)-N(2)-C(6)	113.38(13)
C(8)-N(2)-C(3)	107.62(12)
C(6)-N(2)-C(3)	105.77(12)
C(3)-N(7)-C(5)	105.29(12)

C(3)-N(7)-H(7N)	108.5
C(5)-N(7)-H(7N)	109.9
C(9)-N(10)-C(12)	125.12(14)
C(9)-N(10)-H(10N)	117.3
C(12)-N(10)-H(10N)	116.8
O(2)-C(2)-N(1)	126.37(15)
O(2)-C(2)-C(3)	126.11(15)
N(1)-C(2)-C(3)	107.50(13)
N(7)-C(3)-N(2)	108.52(13)
N(7)-C(3)-C(2)	114.96(13)
N(2)-C(3)-C(2)	104.98(13)
N(7)-C(3)-H(3A)	109.4
N(2)-C(3)-H(3A)	109.4
C(2)-C(3)-H(3A)	109.4
N(7)-C(5)-C(6)	104.09(13)
N(7)-C(5)-H(5A)	110.9
C(6)-C(5)-H(5A)	110.9
N(7)-C(5)-H(5B)	110.9
C(6)-C(5)-H(5B)	110.9
H(5A)-C(5)-H(5B)	109.0
N(2)-C(6)-C(5)	103.91(12)
N(2)-C(6)-H(6A)	111.0
C(5)-C(6)-H(6A)	111.0
N(2)-C(6)-H(6B)	111.0
C(5)-C(6)-H(6B)	111.0
H(6A)-C(6)-H(6B)	109.0
N(1)-C(8)-N(2)	104.74(13)
N(1)-C(8)-C(9)	113.80(13)
N(2)-C(8)-C(9)	110.06(13)
N(1)-C(8)-H(8)	109.4
N(2)-C(8)-H(8)	109.4
C(9)-C(8)-H(8)	109.4
O(1)-C(9)-N(10)	123.42(16)
O(1)-C(9)-C(8)	118.01(14)
N(10)-C(9)-C(8)	118.55(14)
N(10)-C(12)-C(11)	110.26(13)
N(10)-C(12)-H(12A)	109.6
C(11)-C(12)-H(12A)	109.6
N(10)-C(12)-H(12B)	109.6

C(11)-C(12)-H(12B)	109.6
H(12A)-C(12)-H(12B)	108.1
N(1)-C(11)-C(12)	107.87(13)
N(1)-C(11)-H(11A)	110.1
C(12)-C(11)-H(11A)	110.1
N(1)-C(11)-H(11B)	110.1
C(12)-C(11)-H(11B)	110.1
H(11A)-C(11)-H(11B)	108.4

Symmetry transformations used to generate equivalent atoms:

Table S18. Anisotropic displacement parameters (Å^{2}x 10^3) for hexahydroimidazo[1',2':3,4]imidazo[1,2-*a*]pyrazine-5,10(4a*H*,6*H*)-dione (10).

The anisotropic displacement factor exponent takes the form: -2 ^{2}[h^2 a*^{2}U^{11} + ... + 2 h k a* b* U^{12}]

	U^{11}	U^{22}	U^{33}	U^{23}	U^{13}	U^{12}
O(1)	27(1)	15(1)	14(1)	-3(1)	2(1)	0(1)
O(2)	27(1)	20(1)	14(1)	-4(1)	4(1)	0(1)
N(1)	22(1)	13(1)	10(1)	0(1)	0(1)	1(1)
N(2)	20(1)	13(1)	11(1)	1(1)	0(1)	2(1)
N(7)	21(1)	11(1)	16(1)	1(1)	1(1)	1(1)
N(10)	28(1)	12(1)	14(1)	0(1)	1(1)	-3(1)
C(2)	18(1)	13(1)	14(1)	0(1)	1(1)	-4(1)
C(3)	19(1)	12(1)	13(1)	-2(1)	2(1)	-2(1)
C(5)	23(1)	15(1)	17(1)	2(1)	4(1)	0(1)
C(6)	25(1)	15(1)	11(1)	1(1)	2(1)	1(1)
C(8)	19(1)	13(1)	11(1)	-1(1)	1(1)	-1(1)
C(9)	17(1)	14(1)	14(1)	0(1)	-1(1)	2(1)
C(12)	30(1)	15(1)	12(1)	2(1)	1(1)	-2(1)
C(11)	26(1)	18(1)	12(1)	3(1)	-2(1)	0(1)

Table S19. Hydrogen coordinates (x 10^4) and isotropic displacement parameters (Å^{2}x 10 ^{3}) for hexahydroimidazo[1',2':3,4]imidazo[1,2-*a*]pyrazine-5,10(4a*H*,6*H*)-dione (10).

	x	y	z	U(eq)
H(7N)	5385	8156	2947	24
H(10N)	7354	-117	3875	27
H(3A)	4624	5940	3577	17
H(5A)	9008	7452	2949	22
H(5B)	7708	8329	2127	22
H(6A)	6354	5853	1652	21
H(6B)	8388	5224	1967	21
H(8)	9295	4189	3326	17
H(12A)	8029	603	5404	23
H(12B)	6240	1654	5078	23
H(11A)	8362	3521	5794	23
H(11B)	9882	2885	5168	23

Table S20. Torsion angles [°] for hexahydroimidazo[1',2':3,4]imidazo[1,2-*a*]pyrazine-5,10(4a*H*,6*H*)-dione (10).

C(8)-N(1)-C(2)-O(2)	-174.24(15)
C(11)-N(1)-C(2)-O(2)	21.0(3)
C(8)-N(1)-C(2)-C(3)	7.44(19)
C(11)-N(1)-C(2)-C(3)	-157.32(15)
C(5)-N(7)-C(3)-N(2)	-16.25(17)
C(5)-N(7)-C(3)-C(2)	100.91(16)
C(8)-N(2)-C(3)-N(7)	115.35(14)
C(6)-N(2)-C(3)-N(7)	-6.13(17)
C(8)-N(2)-C(3)-C(2)	-8.04(16)
C(6)-N(2)-C(3)-C(2)	-129.52(13)
O(2)-C(2)-C(3)-N(7)	63.2(2)
N(1)-C(2)-C(3)-N(7)	-118.50(15)
O(2)-C(2)-C(3)-N(2)	-177.68(15)
N(1)-C(2)-C(3)-N(2)	0.65(17)
C(3)-N(7)-C(5)-C(6)	31.62(16)
C(8)-N(2)-C(6)-C(5)	-92.44(15)
C(3)-N(2)-C(6)-C(5)	25.25(16)
N(7)-C(5)-C(6)-N(2)	-35.42(16)

C(2)-N(1)-C(8)-N(2)	-12.43(18)
C(11)-N(1)-C(8)-N(2)	153.85(14)
C(2)-N(1)-C(8)-C(9)	-132.67(15)
C(11)-N(1)-C(8)-C(9)	33.6(2)
C(6)-N(2)-C(8)-N(1)	128.66(14)
C(3)-N(2)-C(8)-N(1)	12.06(16)
C(6)-N(2)-C(8)-C(9)	-108.63(15)
C(3)-N(2)-C(8)-C(9)	134.76(13)
C(12)-N(10)-C(9)-O(1)	-177.67(16)
C(12)-N(10)-C(9)-C(8)	4.0(2)
N(1)-C(8)-C(9)-O(1)	176.97(14)
N(2)-C(8)-C(9)-O(1)	59.8(2)
N(1)-C(8)-C(9)-N(10)	-4.6(2)
N(2)-C(8)-C(9)-N(10)	-121.81(16)
C(9)-N(10)-C(12)-C(11)	-28.7(2)
C(2)-N(1)-C(11)-C(12)	106.25(18)
C(8)-N(1)-C(11)-C(12)	-57.97(19)
N(10)-C(12)-C(11)-N(1)	52.17(19)

Symmetry transformations used to generate equivalent atoms:

Table S21. Hydrogen bonds for hexahydroimidazo[1',2':3,4]imidazo[1,2-*a*]pyrazine-5,10(4a*H*,6*H*)-dione (10) [Å and °].

D-H...A	d(D-H)	d(H...A)	d(D...A)	<(DHA)
N(7)–H(7N)···N(2)^{i}	0.90	2.30	3.180(2)	164
N(10)–H(10N)···N(7)^{ii}	0.91	2.05	2.933(2)	165
C(3)–H(3A)···O(1)^{i}	1.00	2.32	3.110(2)	135
C(5)–H(5A)···O(1)^{iii}	0.99	2.59	3.352(2)	133
C(11)–H(11A)···O(1)^{iv}	0.99	2.52	3.463(2)	159

Symmetry codes:

[ii] x,-1+y,z

[i] 1-x,1/2+y,1/2-z

[iii] 2-x,1/2+y,1/2-z

[iv] x,1/2-y,1/2+z

Evaluation of cytotoxicity

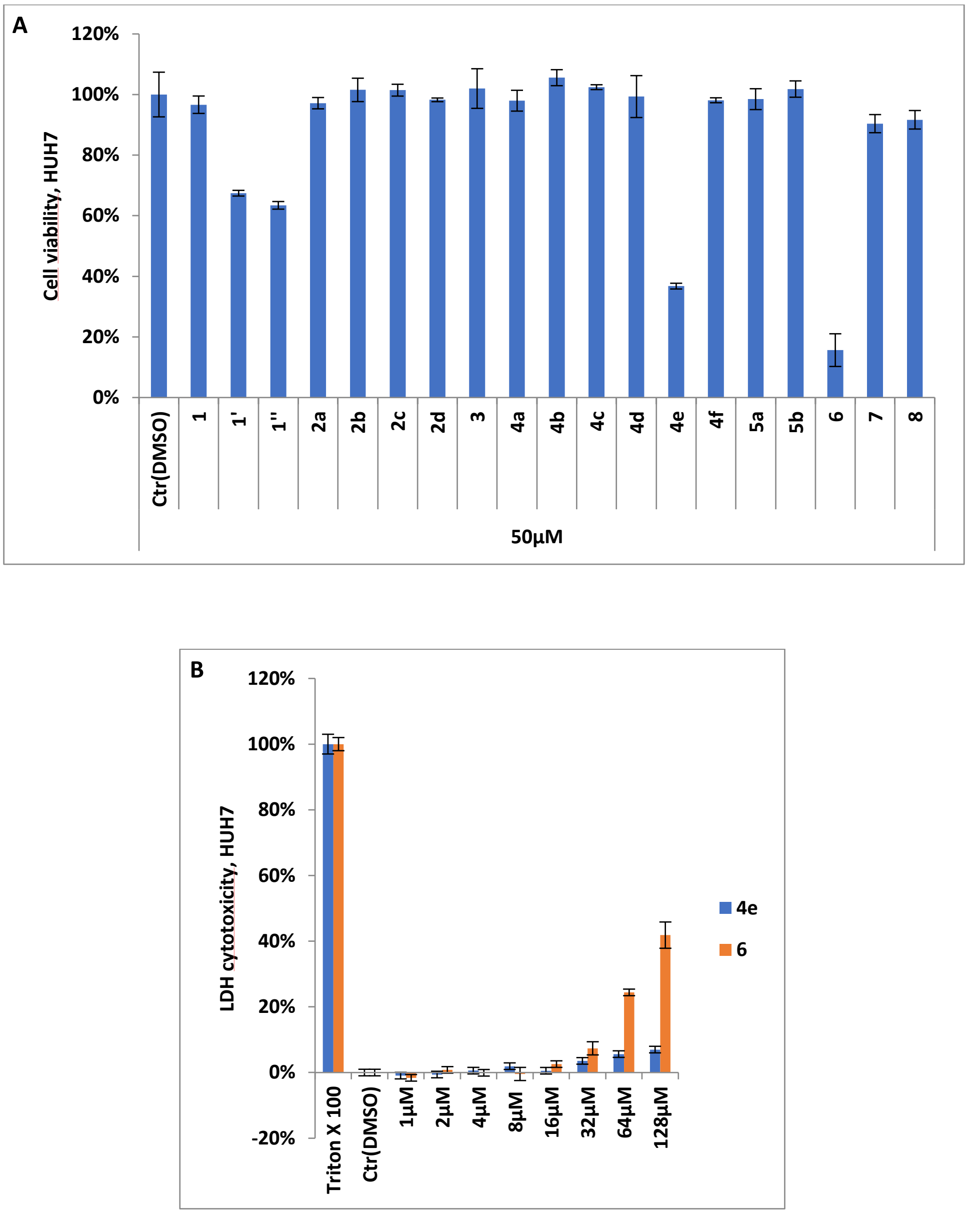

Figure S7. Effect of investigated compounds on HUH7 cell line. (**A**) HUH7 cell line was incubated with 50 µM of the studied compounds or vehicle control (DMSO) for 24 h. (**B**) A quantitative measurement of LDH released into the media for compounds **4e** and **6** after 24 h. We used cell lysis by Triton X 100 as a 100% positive control. All experiments were performed in triplicates, error bars indicate ± SD.

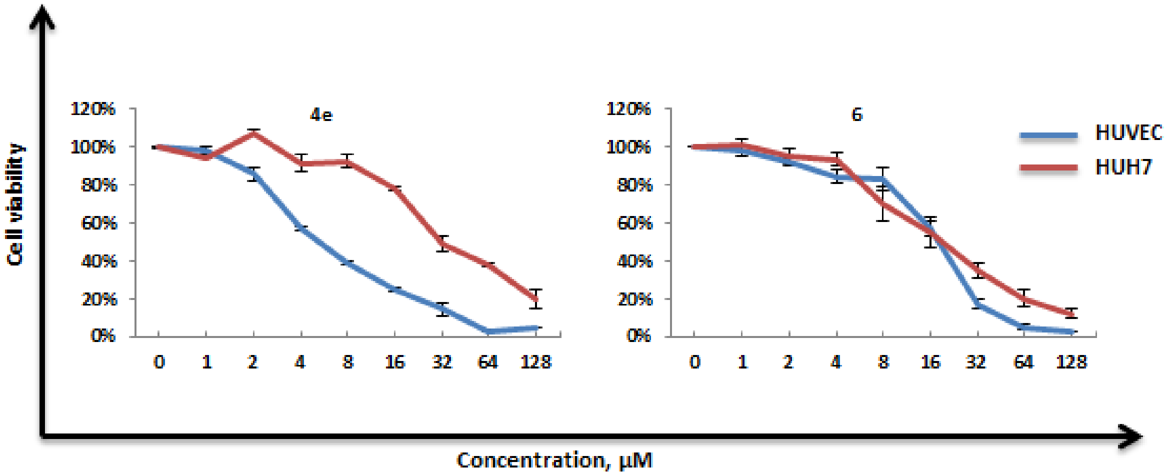

Figure S8. Impact of aminophosphonates **4e** and **6** on HUH7 in contrast to HUVEC. Cell lines are treated with indicated compounds in a dose-dependent manner (1, 2, 4, 8, 16, 32, 64, 128 µM) for 24 h. Values are the mean ±SD of triplicate experiments.

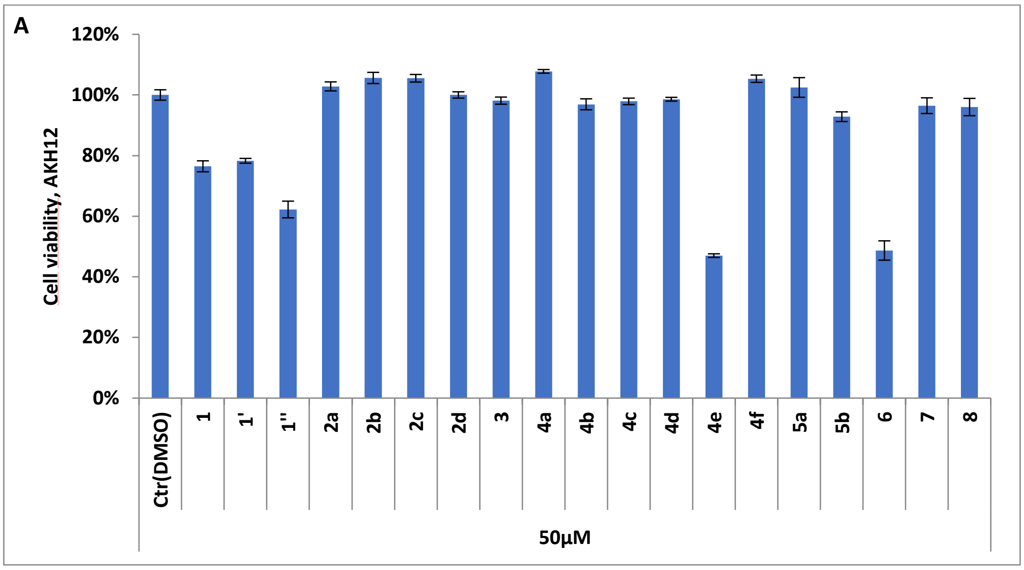

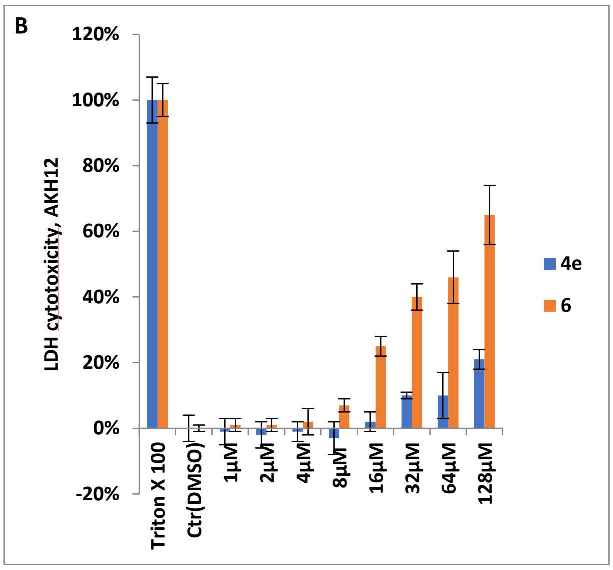

Figure S9.Viability and cytotoxicity effect of investigated compounds on AKH12 cells. (**A**) Cell viability was measured after 24 h of incubation of AKH12 cell line with 50 µM of indicated derivatives. (**B**) LDH release for **4e** and **6** treatment. Triton X 100 was used to induce maximum LDH leakage. All experiments were performed in triplicates; means ± SD errors are shown.

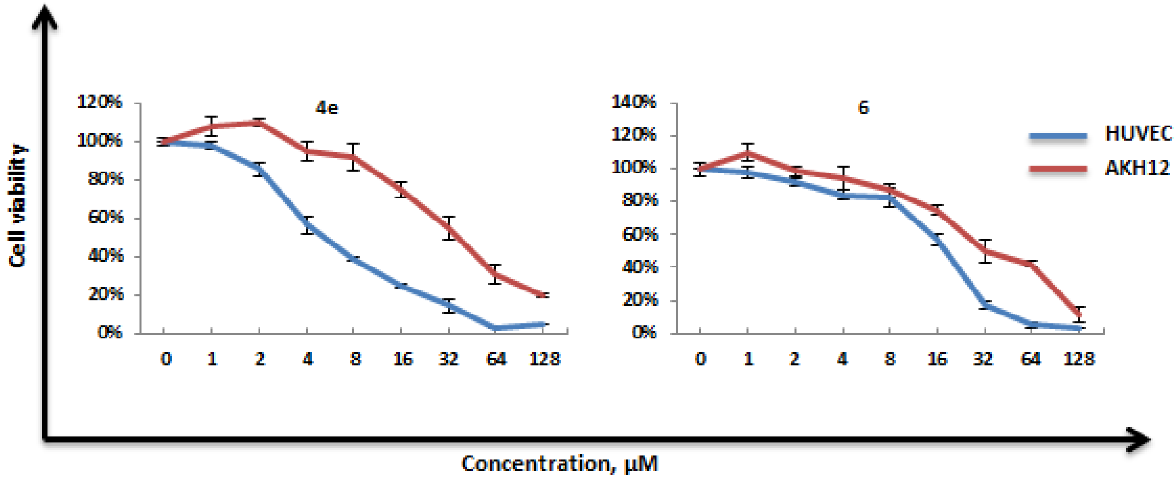

Figure S10. Impact of aminophosphonates **4e** and **6** on AKH12 compared to HUVEC. Cells were treated with indicated aminophosphonates in dose-dependent manner. All experiments were performed in triplicates; means ± SD errors are shown.

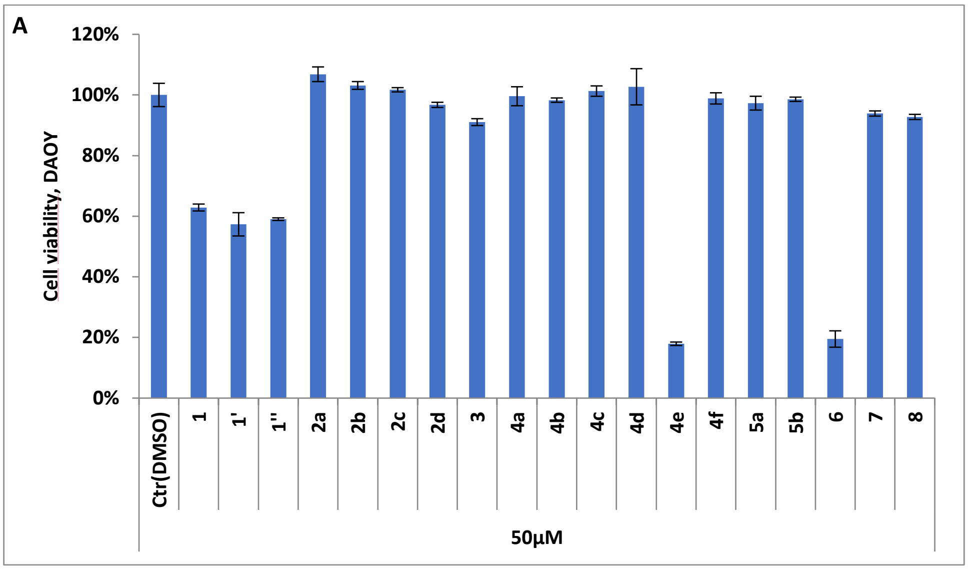

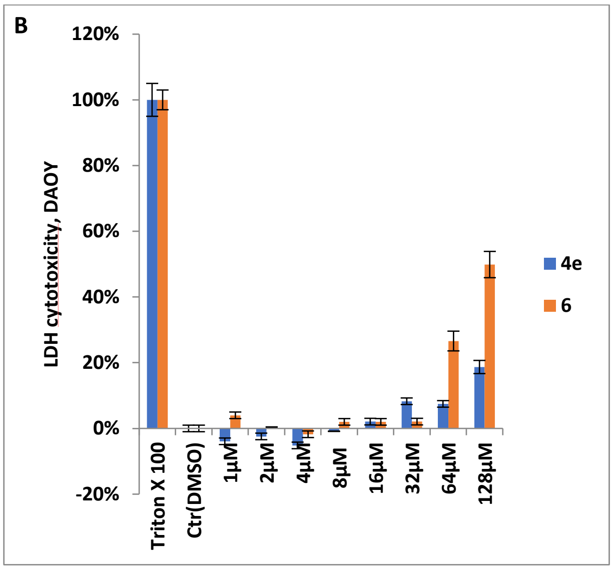

Figure S11. Influence of investigated compounds on DAOY cell viability and cytotoxicity. A) DAOY cells were incubated with 50 µM designated aminophosphonates compounds for 24 h. B) The LDH release assay for 4e and 6. All experiments were performed in triplicates; error bars indicate ± SD.

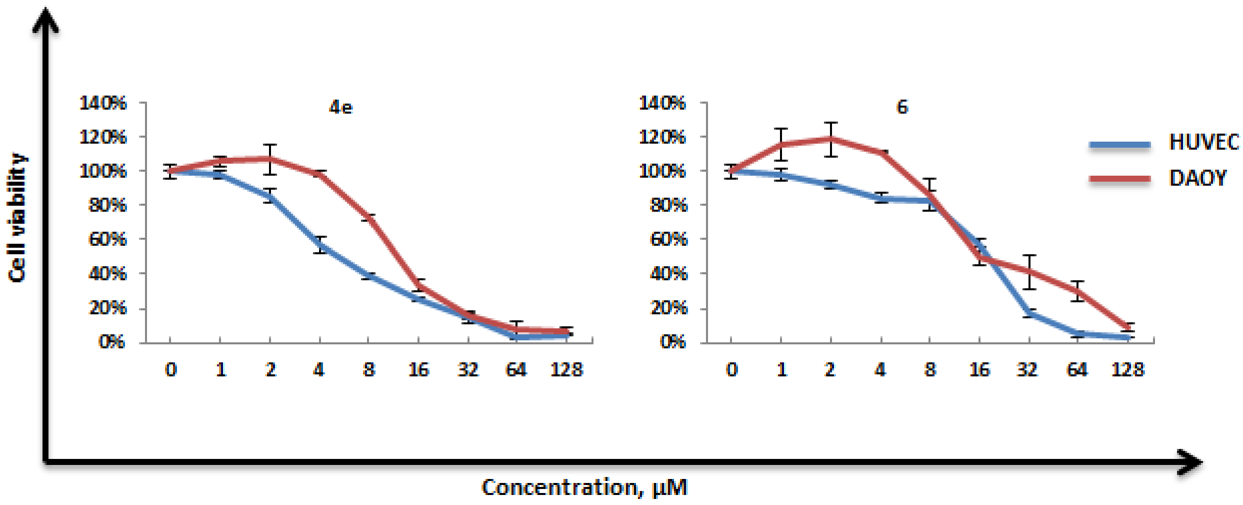

Figure S12. Sensitivity of DAOY to 4e and 6 in contrast to HUVEC cell lines. Error bars indicate mean ± SD.

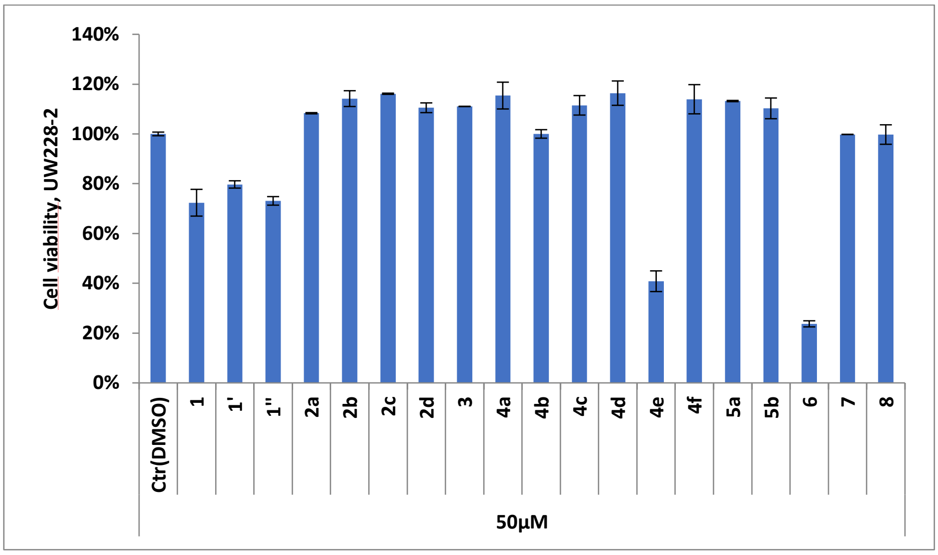

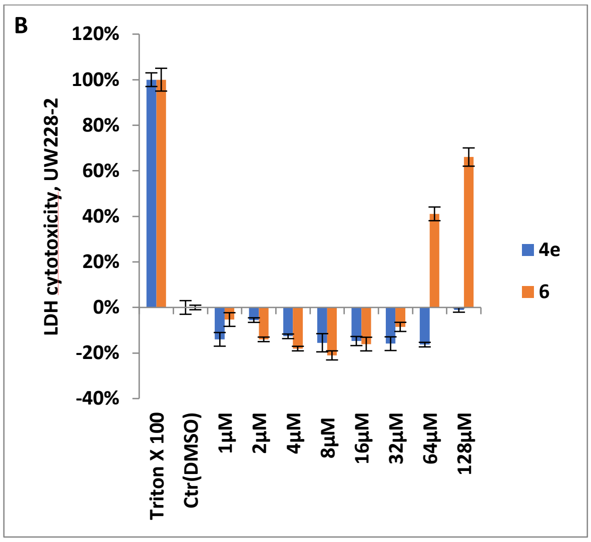

Figure S13. Effect of studied compounds on viability of UW228-2 cell line. A) UW228-2 cells were introduced by 50 μM indicated compounds for 24 h. B) Cytotoxicity of 4e and 6 was determined by LDH release of various concentrations of specified aminophosphonates. All experiments were performed in triplicates; means ± SD errors are shown.

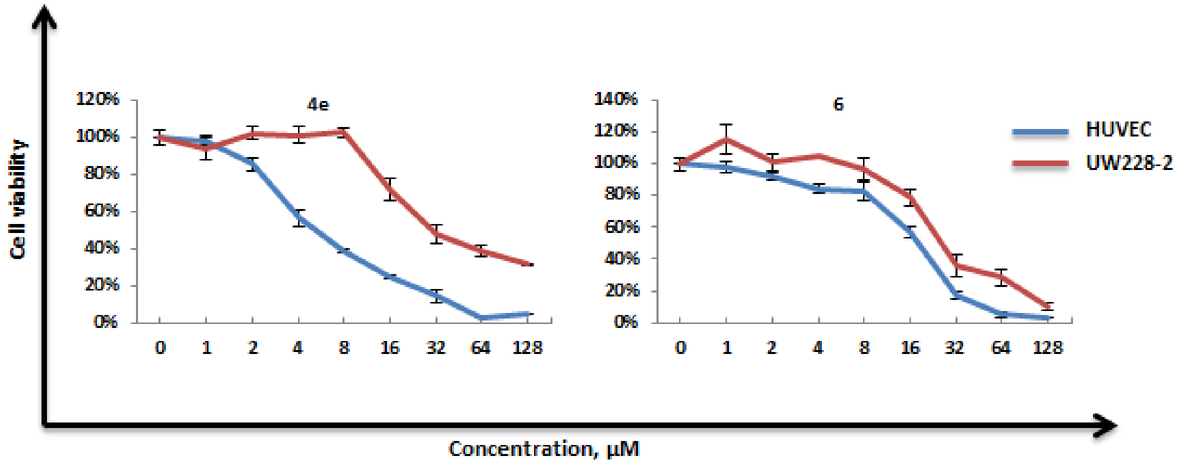

Figure S14. Impact of compounds 4e and 6 on UW228-2 in contrast to HUVEC. Cells treated with a variety of concentration of compounds or DMSO vehicle for 24 h. All experiments were performed in triplicates; errors indicate ± SD.

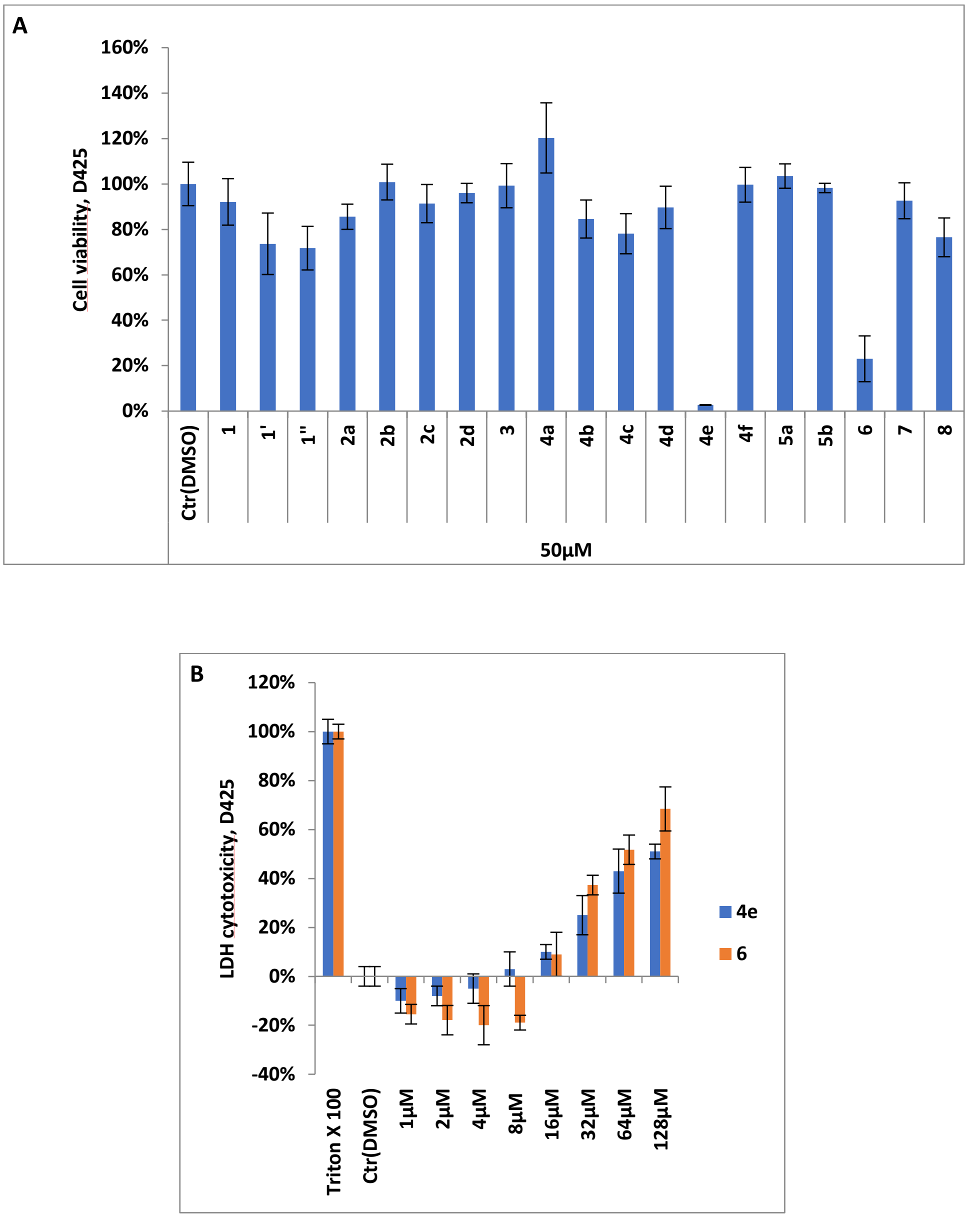

Figure S15. Cytotoxicity of studied compounds on D425 cells. A) D425 cells were incubated with 50 μM of indicated aminophosphonates compounds for 24 h. B) LDH release of compunds 4e and 6 after 24 h in a dose-dependent manner. Triton X 100 used as positive control. Error bars indicate mean ±SD.

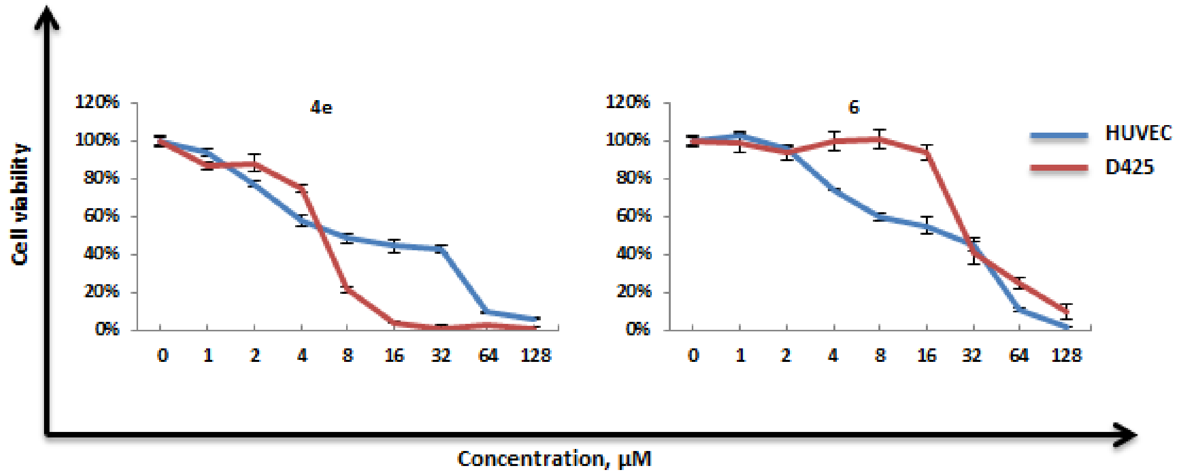

Figure S16. Effect of aminophosphonates 4e and 6 on viability of D425 in comparison with HUVEC cell lines. Grown cells were treated with mentioned compounds in 24-well plates for 24 h. Error bars indicate standard error of mean of triplicate assays.

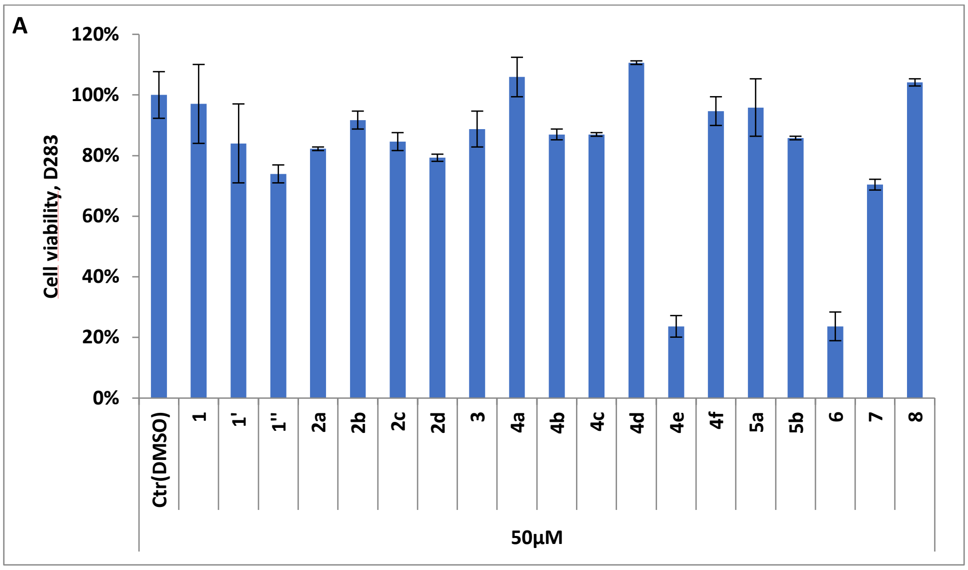

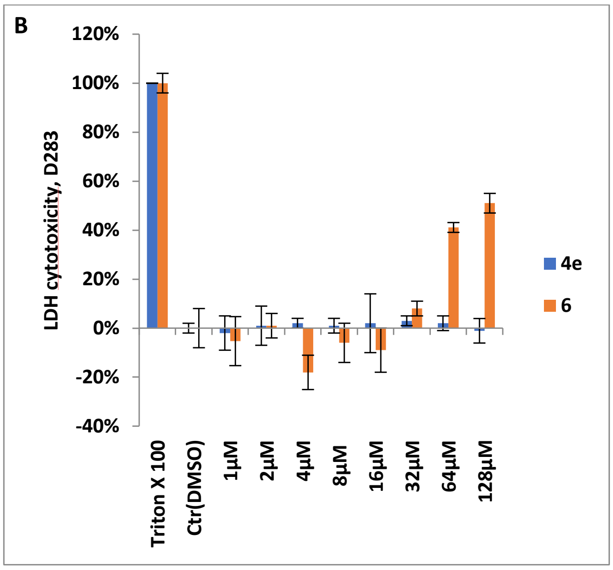

Figure S17. Influence of studied compounds treatment on D283 cell viability and cytotoxicity. A) D283 cells were incubated with 50 µM designated aminophosphonates compounds for 24 h. B) The LDH release assay for selected aminophosphonates 4e and 6. All experiments were performed in triplicates; errors indicate ± SD.

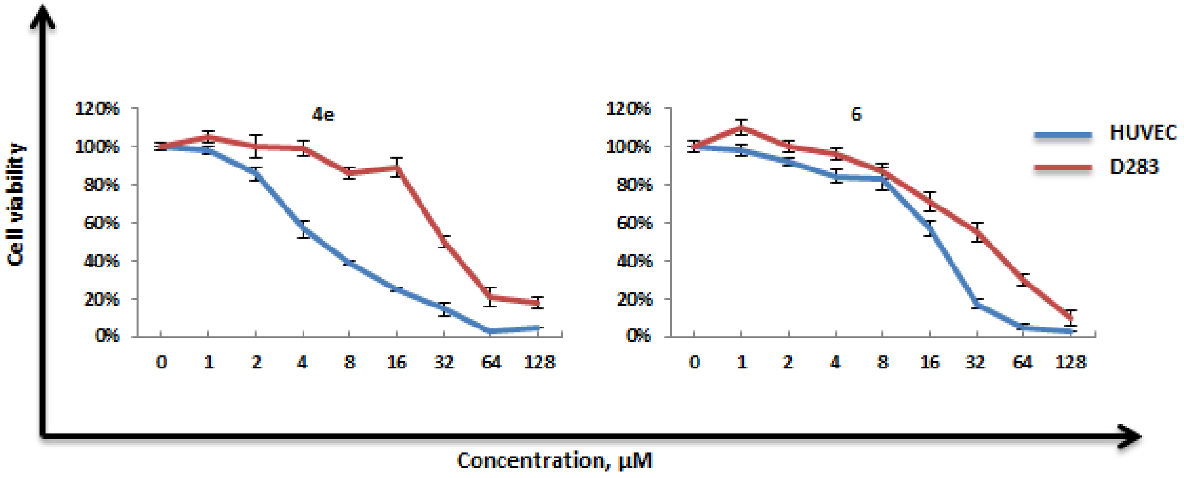

Figure S18. Efficacy of aminophosphonates on cell viability of D283 compared to HUVEC cell lines. Cell lines are treated with indicated compounds in a dose-dependent manner for 24 h. No differences in cytotoxicity between the cell lines were observed. Values are the mean ±SD of triplicate experiments.

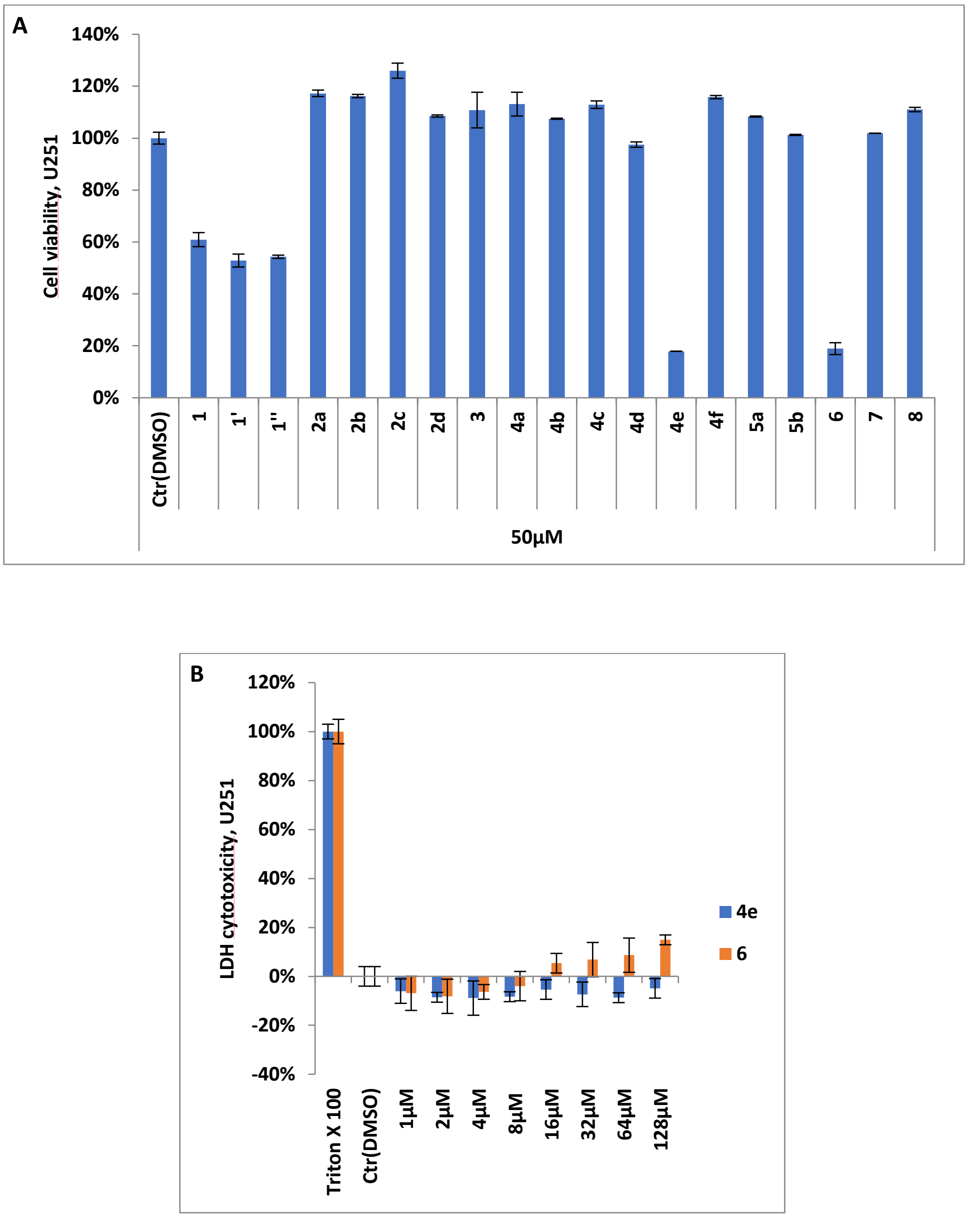

Figure S19. The effect of studied compounds on the viability of U251 cells. A) U251 cell viability was measured after 24 h of 50 μM compounds treatment. B) LDH release. Error bars indicate mean ±SD.

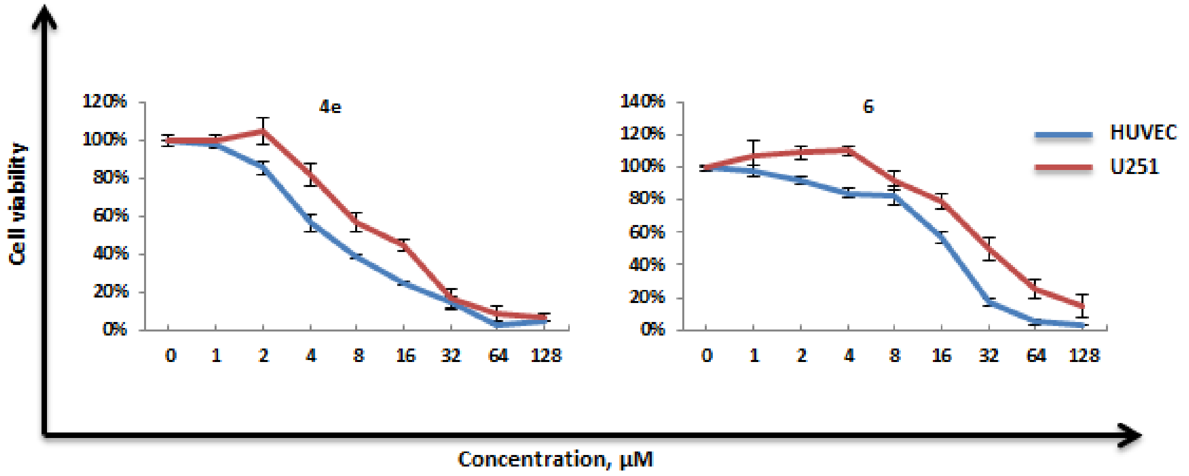

Figure S20. Efficacy of aminophosphonates 4e and 6 on cell viability of U251 compared to HUVEC cell lines. Cell lines are treated with indicated compounds in a dose-dependent manner for 24 h. Values are the mean ±SD of triplicate experiments.
